# Parameter characterization of PEM fuel cell mathematical models using an orthogonal learning-based GOOSE algorithm

**DOI:** 10.1038/s41598-024-71223-7

**Published:** 2024-09-09

**Authors:** Premkumar Manoharan, Sowmya Ravichandran, S. Kavitha, Tengku Juhana Tengku Hashim, Anas R. Alsoud, Tan Ching Sin

**Affiliations:** 1 Department of Electrical and Electronics Engineering, College of Engineering, Institute of Power Engineering (IPE), Universiti Tenaga Nasional (UNITEN), Putrajaya, 43000 Kajang, Selangor Malaysia; 2 Department of Electrical and Electronics Engineering, Dayananda Sagar College of Engineering, Bengaluru, 560078 Karnataka India; 3 Department of Electrical and Electronics Engineering, Manipal Institute of Technology, Manipal Academy of Higher Education, Manipal, 576104 Karnataka India; 4 Department of Electronics and Communication Engineering, M.Kumarasamy College of Engineering, Karur, 639113 Tamil Nadu India; 5 Hourani Center for Applied Scientific Research, Al-Ahliyya Amman University, Amman, Jordan

**Keywords:** Energy, Fuel cells, GOOSE algorithm, Orthogonal learning, PEMFC parameter, Root mean square error, Mathematics and computing, Engineering

## Abstract

In this paper, a new method is designed to effectively determine the parameters of proton exchange membrane fuel cells (PEMFCs), i.e., ${\xi }_{1}$, ${\xi }_{2}$, ${\xi }_{3}$, ${\xi }_{4}$, ${R}_{\text{C}}$, $\lambda$, and $b$. The fuel cells (FCs) involve multiple variable quantities with complex non-linear behaviours, demanding accurate modelling to ensure optimal operation. An accurate model of these FCs is essential to evaluate their performance accurately. Furthermore, the design of the FCs significantly impacts simulation studies, which are crucial for various technological applications. This study proposed an improved parameter estimation procedure for PEMFCs by using the GOOSE algorithm, which was inspired by the adaptive behaviours found in geese during their relaxing and foraging times. The orthogonal learning mechanism improves the performance of the original GOOSE algorithm. This FC model uses the root mean squared error as the objective function for optimizing the unknown parameters. In order to validate the proposed algorithm, a number of experiments using various datasets were conducted and compared the outcomes with different state-of-the-art algorithms. The outcomes indicate that the proposed GOOSE algorithm not only produced promising results but also exhibited superior performance in comparison to other similar algorithms. This approach demonstrates the ability of the GOOSE algorithm to simulate complex systems and enhances the robustness and adaptability of the simulation tool by integrating essential behaviours into the computational framework. The proposed strategy facilitates the development of more accurate and effective advancements in the utilization of FCs.

## Introduction

### Theoretical concepts

DC microgrids are being recognized as a critical component in the future of energy distribution, particularly due to their improved efficiency and stability. The shift is mostly due to the inherent characteristics of DC microgrids with various DC loads and the DC output from various sources, including renewable energy, battery storage, and fuel cells^1,2^. Out of various sources, fuel cells (FCs) are essential components in these DC microgrids. A significant advancement in this field involves the integration of hydrogen and solar energy to establish a reliable and environmentally friendly storage system referred to as hydrogen energy. Hydrogen is widely available and can be found in fossil fuels, water, and many microbes. It is the third most plentiful element on Earth, following silicon and oxygen^3,4^. Despite its abundance, free hydrogen gas does not occur naturally in large quantities except in natural gas reservoirs. Given its potential, the pursuit of hydrogen energy has captured growing international interest. FCs, specifically, are electrochemical devices that convert the chemical energy of hydrogen directly into electricity. The increasing deployment of FCs in transport, portable, and stationary applications underscore their significant benefits: high efficiency, environmentally friendly and quiet operation, and notably high power and energy densities. Currently, the market offers several types of FCs^5^. Notable among these are the microbial fuel cell, solid oxide FC, phosphoric acid fuel cell, alkaline FC, and the proton exchange membrane FC (PEMFC). The PEMFCs are widely recognized for their sophisticated advancement and widespread use, making them a common preference in diverse applications. However, the substantial expenses associated with PEMFCs necessitate the investigation of their operational circumstances. The mathematical model is important for optimizing the performance of PEMFC and minimizing costs by improving modelling procedures. The PEMFC is a complex system that is influenced by multiple factors and demonstrates dynamic and non-linear properties. The system is controlled by a combination of ordinary and/or partial differential equations^5–7^.

In recent years, various semi-empirical models have been developed to represent the complex behaviours of PEMFCs accurately. Foundational works by^8–11^ have significantly contributed to the development of these models, providing critical insights into the electrochemical and thermodynamic processes within PEMFCs. The accurate modelling of PEMFCs is essential in order to understand the operating mechanisms of FC. The accurate modelling saves time and effort and also improves operational efficiency. The polarization curve is a crucial component of PEMFC modelling since it demonstrates the relation between the output voltage and the current and summarizes the FC's behaviour under different operating conditions^12,13^. Despite research efforts to develop accurate models for extracting PEMFC parameters, the problems remain unsolved. Since the information in the datasheets is insufficient, it is normal to observe differences between the predicted data and the actual data provided by manufacturers^14,15^. Parameter estimation is a dynamic problem where the optimal solution is achieved through the many heuristic and metaheuristic methods. Conventional optimization algorithms lack accuracy and precision when solving the non-linear characteristics of PEMFC. On the other hand, metaheuristic algorithms begin with a randomly chosen estimate and are capable of converging towards a globally optimal solution, successfully solving complex optimization problems^16–19^. The ability of the metaheuristic algorithms to adjust and withstand difficult conditions is beneficial when handling the complicated landscape of estimating parameters in PEMFC. The researchers have developed a model for estimating the parameters that attain high accuracy and efficiency. The original mathematical model guides the design and integration of FCs, and it also offers an understanding of the physical phenomena. The FC electrochemical models strongly depend on experimental data and empirical formulations and it highlights the need for new and adaptable modelling to represent the dynamics of FC operations accurately^20–22^.

### Literature review

Recently, the researchers started utilizing metaheuristic algorithms for deriving the unknown parameters of the PEMFC model. The precision and efficiency of such models have increased as a result of advances in intelligence approaches, which have been responsible for the transformation^23–25^. Metaheuristic algorithms have developed as effective and reliable methods for estimating PEMFC parameters, as discussed in various literature^26–28^. Traditional algorithms, such as particle swarm optimization (PSO)^30^ and genetic algorithm (GA)^29^ have been used in various real-time applications. Nevertheless, traditional methods often suffer from low computational efficiency and a dependency on initial conditions, limiting their ability to locate optimal solutions. The advanced algorithms include a variety of algorithms such as the shark smell algorithm^31^, coyote optimization algorithm^32^, beluga whale optimization algorithm^33^, grey wolf optimizer^34,35^, whale optimization algorithm^36,37^, grasshopper optimization algorithm^38^, moth flame optimizer^39^, bald eagle search optimizer^40^, bonobo algorithm^41^, Newton–Raphson-based algorithm^42^, manta ray forage optimization^43^, pathfinder algorithm^44^, reptile search algorithm^45,46^, harris hawk algorithm^47^, golden jackal algorithm^48,49^, jellyfish algorithm, black widow algorithm^50^, artificial ecosystem optimization^51^, artificial rabbits optimizer^52,53^, tree-seed algorithm^54^, mountain gazelle optimizer^55,56^, neural network algorithm^23^, tree-growth algorithm^57^, gradient-based optimizer^14,58,59^, sparrow search algorithm^60,61^, flower pollination method^62^, resistance–capacitance algorithm^63,64^, political optimizer^19,65^, exponential distribution algorithm^66,67^, marine predator algorithm^68,69^, and slime mould optimization algorithm^70^ have also been applied to this domain. In more specific studies, a combination of a teaching learning-based optimizer and differential evolution approach has been developed alongside a modified salp swarm optimizer aimed at investigating optimal PEMFC stack parameters^71,72^. To reduce the shortcomings of the algorithms mentioned above, several improved algorithms have been proposed in different literature. For instance, the authors of^73^ have introduced an improved differential evolutionary optimization algorithm, enhancing search efficiency and control parameter sensitivity through an adaptive method. Recent developments include the salp swarm algorithm^74^, enhanced fluid search optimization algorithm^75^, LSHADE-EpSin algorithm^76^, improved artificial ecosystem optimization^77^, etc., are also reported to estimate the parameters of the PEMFC model and each algorithm offers benefits in terms of precision, convergence rate, and computational burden. Furthermore, the Bayesian regularized neural network is employed for extracting PEMFC parameters along with the improved barnacles mating optimization^78^ and dynamic sparrow search optimizer^79^. Notably, the authors of^80^ proposed a heap-based optimizer for PEMFC parameter identification. Additionally, atom search optimization algorithms^81^ and Harris Hawks’ optimization^82^ and have been utilized for similar purposes, with the authors of^83^ proposing a balanced version of the slime mould algorithm for enhanced performance. Lastly, chaos-embedded PSO has also been utilized for parameter estimation, showcasing the dynamic and evolving landscape of PEMFC parameter identification through metaheuristic algorithms^84^.

### Purpose and significance

This paper introduces an estimation algorithm that utilizes the Orthogonal Learning-based GOOSE (OLGOOSE) algorithm, an advanced meta-heuristic inspired by the unique foraging and resting behaviour of geese. The proposed GOOSE algorithm increased the exploitation capabilities and rapid convergence, making it highly effective for complex multi-modal optimization problems. The proposed method estimates critical variables ${\xi }_{1},{\xi }_{2},{\xi }_{3},{\xi }_{4},\lambda ,b,{R}_{c}$ in the adopted electrical fuel cell model. While these parameters are specific to our model, the OLGOOSE algorithm proposed can be adapted to estimate parameters in other empirical or semi-empirical models, as evidenced by the works of^8–11^. The purpose of this study is to develop a robust method for accurately determining the parameters of PEMFCs using the OLGOOSE algorithm. The study aims to enhance the precision of parameter estimation by integrating an orthogonal learning mechanism, which improves the optimization process and leads to more accurate models. The study also aims to compare the performance of the OL-GOOSE algorithm with existing methods, such as the original GOOSE algorithm and other advanced algorithms, to validate its effectiveness. Additionally, the study seeks to address the issue of computational efficiency by ensuring that the proposed method reduces the time required for parameter estimation, making it feasible for real-time applications. Another goal is to demonstrate the reliability of the OLGOOSE algorithm, which will be evaluated through comprehensive experimental data analysis.

The significance of this study lies in its potential to improve the accuracy and reliability of PEMFC models, which are crucial for optimizing fuel cell performance and durability. By providing a more precise method for parameter estimation, the study contributes to the effective design, optimization, and operational control of PEMFCs. The proposed OLGOOSE algorithm is adaptable and can be applied to various empirical and semi-empirical models, broadening its applicability and making it a valuable tool for researchers and engineers. The study also enhances computational efficiency, enabling the use of the algorithm in real-time scenarios and large-scale simulations. Furthermore, by improving the reliability of PEMFC models, the study supports advancements in automotive applications, stationary power generation, and portable power devices, contributing to the broader field of clean energy technology. Finally, the integration of orthogonal learning mechanisms in optimization algorithms presented in this study can inspire similar advancements in other complex engineering systems, thereby extending its impact beyond PEMFCs.

### Addressing research gaps with OLGOOSE

The authors reviewed existing studies on parameter identification for PEMFCs and found several limitations. Many current methods struggle to achieve high accuracy due to the complex and non-linear nature of PEMFCs. These methods often require extensive computational resources and time, making them impractical for real-time applications or large-scale simulations. Additionally, the reliability of the parameter estimates can vary significantly, leading to less robust models. Furthermore, some methods are highly specific to certain types of PEMFC models and do not generalize well to other models or conditions. The proposed OLGOOSE algorithm addresses these gaps in several ways. First, by integrating an orthogonal learning mechanism, the OLGOOSE algorithm enhances the optimization process, resulting in more precise parameter estimates. This improvement in accuracy is crucial for developing reliable PEMFC models. Second, the OLGOOSE algorithm is designed to be computationally efficient, reducing the time required for parameter estimation and making it appropriate for real-time claims and large-scale simulations. Third, the adaptive behaviour and robust optimization framework of the OLGOOSE algorithm increase the reliability of the parameter estimates, ensuring consistent and dependable performance under various operating conditions. Finally, the versatility of the OLGOOSE algorithm allows it to be applied to various empirical and semi-empirical PEMFC models, making it a valuable tool for researchers and engineers working with different types of fuel cell systems and conditions.

The main contributions of this paper are outlined as follows:

- Proposes a robust methodology employing the OLGOOSE algorithm to estimate the most accurate parameters of PEMFC models.
- Demonstrates the dominance of the OLGOOSE algorithm in finding the optimal parameters for various PEMFC models, showcasing its enhanced performance compared to traditional methods.
- Checking the reliability and strength of the OLGOOSE, the convergence curve, I-V, and P–I curves are obtained.
- Provide a comprehensive comparison of the OLGOOSE algorithm with other prominent optimization techniques.

The structure of this paper is organized into several parts for clarity and depth: Section "Modelling and problem formulation" details the model of the fuel cell. Section "Modelling and problem formulation" also delves into mathematical modelling, including the definition of the objective function. Section "Orthogonal learning based GOOSE algorithm" describes the implementation of the proposed OLGOOSE algorithm used in this study. Section "Results and discussions" examines the performance of the proposed strategy through empirical testing and also presents a comparison of the results obtained with those from recently published algorithms. The paper concludes with Section "Conclusions", where the findings are summarized and potential future work is discussed.

## Modelling and problem formulation

**Fig. 1:**
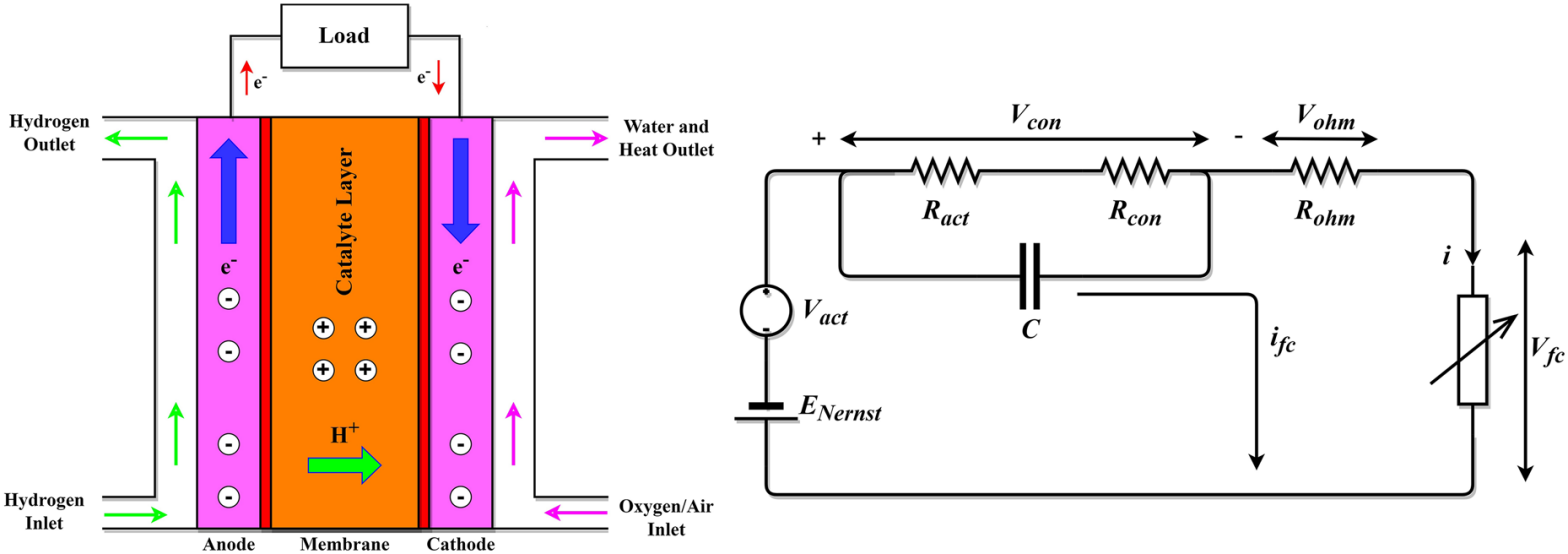
Structure of the PEMFC and the equivalent circuit.

Building upon the foundational semi-empirical models by^8–11^, this study proposes an improved parameter estimation procedure. In a PEMFC, the core components are the anode and cathode, which are separated by a polymer electrolyte membrane, as depicted in Fig. 1. The operational mechanics involve hydrogen being introduced at the anode side while oxygen is fed into the cathode. The polymer electrolyte membrane serves a dual role: it conducts ions between the two electrodes and acts as a barrier to electron flow, ensuring that electrons must travel through an external circuit, thus generating electrical output^85^. The PEMFC functions based on a series of electrochemical reactions. At the anode, hydrogen molecules are split into protons and electrons. The polymer membrane allows protons to pass through to the cathode, but electrons are forced to travel around the external circuit, creating an electric current. At the cathode, these electrons recombine with the protons and oxygen to form water, completing the chemical process^86^. At the anode, the reaction is represented by Eq. (1), and it describes the separation of hydrogen molecules into protons and electrons.

1 $${\text{H}}_{2}\to 2{\text{H}}^{+}+2{\text{e}}^{-}$$

Equation 2 provides the reaction at the cathode, and it illustrates the reduction of oxygen and the combination with protons and electrons to form water.

2 $${\text{O}}_{2}+4{\text{H}}^{+}+4{\text{e}}^{-}\to 2{\text{H}}_{2}O$$

Equation 3 gives the chemical reaction that represents the total electrical energy generation.

3 $$2{\text{H}}_{2}+{\text{O}}_{2}\to 2{\text{H}}_{2}\text{O}$$

This reaction summarizes the complete process occurring within the PEMFC, from hydrogen oxidation at the anode to water formation at the cathode. These reactions underscore the continuous movement of ions and electrons within the fuel cell, which is essential for the production of electricity, thereby highlighting the complex yet efficient nature of PEMFCs in energy conversion^24^. The electrical generation is given by Eq. (4).

4 $${V}_{fc}={E}_{Nernst}-{V}_{act}-{V}_{ohm}-{V}_{con}$$

In the operation of an FC, the terminal voltage ${V}_{fc}$ is composed of several distinct components, each representing different types of voltage losses, as outlined in Eq. (4). These components are crucial for understanding how an FC converts chemical energy into electrical energy efficiently: (i) ${E}_{Nernst}$ is the adjustable open-circuit voltage, which is the ideal value that the fuel cell would produce if there were no losses due to the cell's operation; (ii) ${V}_{act}$ is the activation voltage drop, this component arises from the energy barrier that must be overcome to initiate the electrochemical reactions at the electrodes. ${V}_{act}$ includes the activation losses from both the anode and the cathode. At the anode, hydrogen molecules are oxidized, as described by Eq. (1), while at the cathode, oxygen molecules are reduced, as described by Eq. (2). The overall reaction for the PEMFC, which results in electricity generation, is provided in Eq. (3). This comprehensive reaction highlights the continuous ion and electron movement essential for the efficient energy conversion process in PEMFCs; (iii) ${V}_{ohm}$ is the ohmic voltage drop, which occurs due to the resistance to the flow of ions through the electrolyte and the resistance to the flow of electrons in the external circuit; (iv) ${V}_{con}$ represents the concentration voltage drop, which happens when there are variations in the concentration of reactants at the electrode surfaces. Together, these components determine the actual operating voltage of the FC, highlighting the various inefficiencies that can occur during its operation. The expression for ${E}_{Nernst}$ is provided in Eq. (5).

5 $${E}_{\text{Nernst}}={V}_{oc}={E}_{r}\left(T,P\right)+\frac{R{T}_{c}}{ZF}\left[\text{ln}\left({P}_{{H}_{2}}\right)+\text{ln}\left(\sqrt{{P}_{{O}_{2}}}\right)\right]$$

6 $${E}_{r}\left(T,P\right)={E}_{0}+\frac{\Delta G}{ZF}$$

where ${E}_{0}$ is the standard reference voltage at standard conditions, $\Delta G$ is the change in Gibbs free energy, which is a function of temperature and pressure, $Z$ is the number of electrons transferred in the reaction, and $F$ is the Faraday constant, which represents the electric charge per mole of electrons. ${E}_{r}$ represents the reference voltage (standard cell potential) utilizing total Gibbs free energy and it is indeed a function of reaction pressure and temperature, and fluctuations in these parameters can significantly impact the reference voltage. The dependency of ${E}_{r}$ on temperature and pressure is given by ${E}_{r}\left(T,P\right)$. The reversible thermodynamic potential for the reaction between oxygen and hydrogen in a fuel cell is determined by the Nernst equation, as illustrated in Eq. (6). In this equation, $R$ signifies the universal gas coefficient, ${T}_{c}$ indicates the cell temperature in K and ${P}_{{H}_{2}}$ and ${P}_{{O}_{2}}$ are the partial pressures of hydrogen and oxygen, respectively. Equation (5) can be rewritten to account for these variables in a more detailed form explicitly.

7 $${E}_{\text{Nernst}}=1.229-8.5\times {10}^{-4}\left({T}_{c}-298.15\right)+4.385\times {10}^{-5}{T}_{c}\times \left(\text{ln}\left({P}_{{H}_{2}}\right)+\text{ln}\left(\sqrt{{P}_{{O}_{2}}}\right)\right)$$

The Nernst potential ${E}_{\text{Nernst}}$ is expressed as a function of temperature and partial pressures of hydrogen and oxygen. Equation (7) incorporates the standard reference potential (1.229 V) and adjusts for temperature variations with the term $-8.5\times {10}^{-4}\left({T}_{c}-298.15\right)$. The pressure dependencies are reflected in the logarithmic terms involving ${P}_{{H}_{2}}$ and ${P}_{{O}_{2}}$, scaled by $4.385\times {10}^{-5}{T}_{c}$. This formulation ensures an accurate representation of the Nernst potential under varying reaction pressures and temperatures. The partial pressures of oxygen and hydrogen within a PEMFC are detailed in Eqs. (8) and (9), respectively.

8 $${P}_{{O}_{2}}=\left({PH}_{cathode}\times {P}_{{H}_{2}O}\right)\left[{\left(exp\left(\frac{4.192\left(\frac{i}{A}\right)}{{{T}_{c}}^{1.334}}\right)\times \frac{\left({PH}_{anode}\times {P}_{{H}_{2}O}\right)}{{P}_{cathode}}\right)}^{-1}-1\right]$$

9 $${P}_{{H}_{2}}=0.5\left({PH}_{anode}\times {P}_{{H}_{2}O}\right)\left[{\left(exp\left(\frac{1.635\left(\frac{i}{A}\right)}{{{T}_{c}}^{1.334}}\right)\times \frac{\left({PH}_{anode}\times {P}_{{H}_{2}O}\right)}{{P}_{anode}}\right)}^{-1}-1\right]$$

where ${PH}_{anode}$ and ${PH}_{cathode}$ represent the partial pressures of hydrogen and oxygen at the anode and cathode inputs, respectively, ${P}_{anode}$ and ${P}_{cathode}$ represent the total pressures at the anode and cathode sides, respectively, ${P}_{{H}_{2}O}$ represents the partial pressure of water vapour within the system, $i$ represents the electrical current density produced by the PEMFC, and $A$ denotes the membrane’s surface area, both of which play crucial roles in the overall functionality of the cell. The partial pressure of water vapour within the system is elaborated in Eq. (10). The partial pressure of water within the system is determined using the following equation.

10 $${\text{log}}_{10}\left({P}_{{H}_{2}o}^{sat}\right)=2.95\times {10}^{-2}\times \left({T}_{c}-273.15\right)-9.19\times {10}^{-5}\times {\left({T}_{c}-273.15\right)}^{2}+1.44\times {10}^{-7}{\left({T}_{c}-273.15\right)}^{3}-2.18$$

**Fig. 2:**
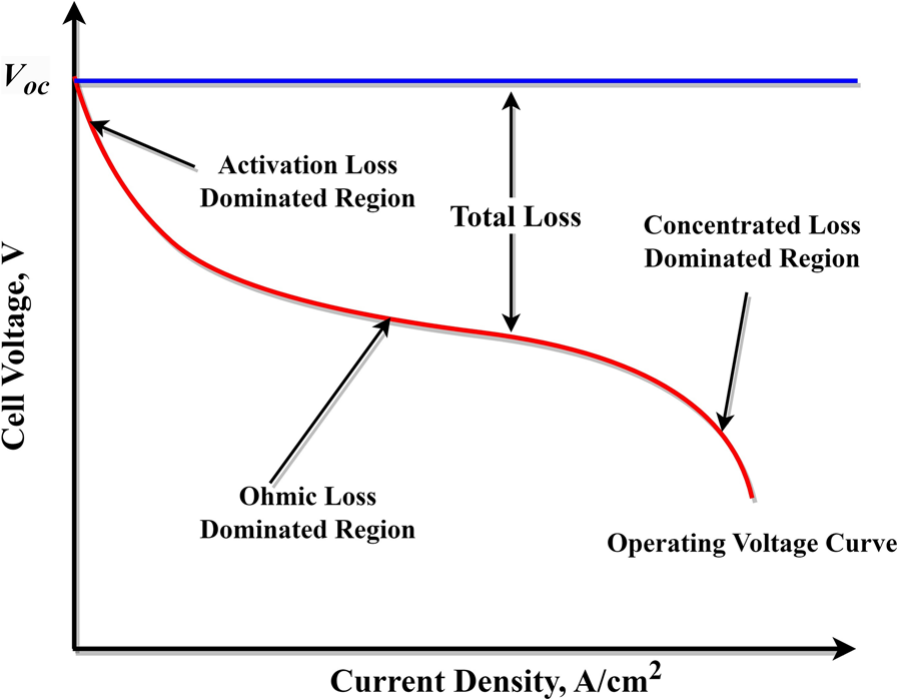
Polarization curve of the PEMFC stack.

Figure 2 displays the simulated current–voltage (I-V) characteristics of a single cell within a PEMFC stack based on the input fuel pressures. When the current level is low, the ohmic loss is less significant because the chemical reactions occurring at the electrode surface proceed at a slower rate. The activation potential $\left({V}_{\text{act}}\right)$ characterizes this reduced rate of reaction, a phenomenon which occurs in what is known as the active polarization region. The formula to calculate this overall activation potential is provided in Eq. (11).

11 $${V}_{act}=-[{\xi }_{1}+{\xi }_{2}T+{\xi }_{3}T\text{ln}\left({C}_{{O}_{2}}\right)+{\xi }_{4}T\text{ln}\left(i\right)]=\frac{R{T}_{c}}{\alpha F}\text{ln}\left(\frac{i}{{i}_{0}}\right)$$

where ${\xi }_{1}$, ${\xi }_{2}$, ${\xi }_{3}$, and ${\xi }_{4}$ are the semi-empirical factors, ${i}_{0}$ signifies the exchange current density, α is the charge transfer factor, and $i$ is the current density. The oxygen concentration ${C}_{{O}_{2}}$ at the interface of catalyst and cathode is provided in Eq. (12).

12 $${C}_{{O}_{2}}=\frac{{P}_{{O}_{2}}}{5.08\times {10}^{6}\times {exp}^{\left(-498/T\right)}}$$

The ohmic region in an FC is characterized by a linear slope that lies between the concentration and the active regions. This region is defined by the losses that occur due to the resistance faced by electrons passing through the external circuit and ions moving through the electrolyte. These resistances lead to a direct and linear relationship between the voltage drop and the current. Consequently, the ohmic loss, denoted as ${V}_{\text{ohm}}$, is expressed in Eq. (13).

13 $${V}_{ohm}=i\cdot ({R}_{m}+{R}_{c})$$

where, ${R}_{m}$ represents the electronic resistance while ${R}_{c}$ refers to the contact resistance or ionic resistance measured in $\left(\Omega { \, {\text{cm}}}^{-2}\right)$. The value of ${R}_{m}$, presented in Eq. (14), varies with minor changes in current or voltage and is a typical resistance characteristic.

14 $${R}_{m}={\rho }_{m}\left(\frac{l}{A}\right)$$

15 $${\rho }_{m}=\frac{181.6\left(1+0.03\left({i}_{c}/A\right)+0.062{\left(T/303\right)}^{2}{\left(i/A\right)}^{2.5}\right)}{\left(\lambda -0.634-3\left(\frac{i}{A}\right)\right)exp\left(4.18\left(\frac{T-303}{T}\right)\right)}$$

where $\lambda$ represents the membrane water content, which is influenced by the stoichiometric ratio of the feed gas at the anode and the relative humidity. Additionally, ${\rho }_{m}$ signifies the membrane-specific resistivity, measured in $\left(\Omega \cdot \text{cm}\right)$. When the current density is extremely high, a notable voltage drop occurs due to the diminished efficiency of gas exchange, often caused by water flooding the catalyst and it is identified as the concentration region. The associated voltage loss, referred to as both mass transport loss and concentration loss, is calculated using Eq. (16).

16 $${V}_{Con}=-b\cdot \text{ln}\left(1-\frac{i}{{i}_{max}}\right)$$

where $i$ represents the actual current density and ${i}_{max}$ denotes the maximum achievable current density, measured in $\left(\text{A}/{\text{cm}}^{2}\right)$. The parameter $b=R{T}_{c}/F$ is a voltage coefficient whose value varies depending on the different working conditions of the FC. The schematic circuit illustrated in Fig. 1 includes the equivalent concentration resistance $\left({R}_{\text{Con}}\right)$ and the equivalent activation resistance $\left({R}_{\text{act}}\right)$. The voltage drops across ${R}_{\text{act}}$ and ${R}_{\text{act}}$ is denoted as ${V}_{Con}$. In order to reduce variability in ${V}_{Con}$, a capacitance $C$ is utilized, which also demonstrates an Electrochemical double-layer capacitance effect as a result of the arrangement of electrodes and the membrane. ${E}_{\text{Nernst}}$ denotes the Nernst voltage, which is also referred to as the thermal or open circuit voltage. The utilization of this analogous circuit is essential for examining the consistent state and active characteristics of the fuel cell. The FC numerical model integrates computational dynamics to tackle the details of charge transport, multidimensional mass and electrochemical kinetics, all of which are interconnected in a temperature-dependent manner. These aspects provide complex difficulties that can be effectively tackled by modern algorithms that can provide precise outcomes with efficient convergence rates. The performance of PEMFCs is highly dependent on efficient water management. Inadequate management can result in significant flooding, causing a substantial increase in the current density within flooded regions, up to 4.7 times larger than in non-flooded areas, resulting in extensive degradation of the FC's performance.

Combining all these elements, it is possible to get the relationship between the cell voltage (${V}_{fc}$) and current density ($j$):

17 $${V}_{fc}={E}_{r}+\frac{R{T}_{c}}{ZF}\left[\text{ln}\left({P}_{{H}_{2}}\right)+\text{ln}\left(\sqrt{{P}_{{O}_{2}}}\right)\right]-\frac{RT}{\alpha F}\text{ln}\left(\frac{i}{{i}_{0}}\right)-i\cdot ({R}_{m}+{R}_{c})-\frac{RT}{F}\text{ln}\left(1-\frac{i}{{i}_{max}}\right)$$

For practical purposes and clarity in presenting the polarization curve, Eq. (16) can be simplified and rearranged as follows.

18 $$V_{fc} = V_{oc} - \frac{RT}{{\alpha F}}\ln \left( {\frac{i}{{i_{0} }}} \right) - i \cdot \left( {R_{m} + R_{c} } \right) - b \cdot \ln \left( {1 - \frac{i}{{i_{max} }}} \right)$$

Equation (17) demonstrates how the actual operating voltage of the PEMFC decreases from the open-circuit voltage ${V}_{oc}$ as the current density increases due to various losses, it encapsulates the relationship between the cell voltage and current density, providing a comprehensive understanding of the PEMFC's performance characteristics.

The semi-empirical modelling of PEMFC illustrates how its performance is influenced by concentration, ohmic, and activation losses. The activation potential is determined by semi-empirical parametric coefficients, denoted as ${\xi }_{1}$, ${\xi }_{2}$, ${\xi }_{3}$, and ${\xi }_{4}$. Ohmic losses are influenced by the ionic resistance and a scalar factor called $\lambda$, which ranges in value from 10 to 24. If the value of $b$ is known, it is possible to calculate the concentration voltage, along with values of current density $J$ and maximum current density $\left({J}_{\text{max}}\right)$. Therefore, for accurate PEMFC modelling, it is essential to determine seven key parameters: ${\xi }_{1}$, ${\xi }_{2}$, ${\xi }_{3}$, ${\xi }_{4}$, ${R}_{\text{C}}$, $b$, and $\lambda$. Such variables are typically not specified in the manufacturer's datasheet and vary under diverse working circumstances, affecting the PEMFC's performance as observed in its polarization curve.

**Fig. 3:**
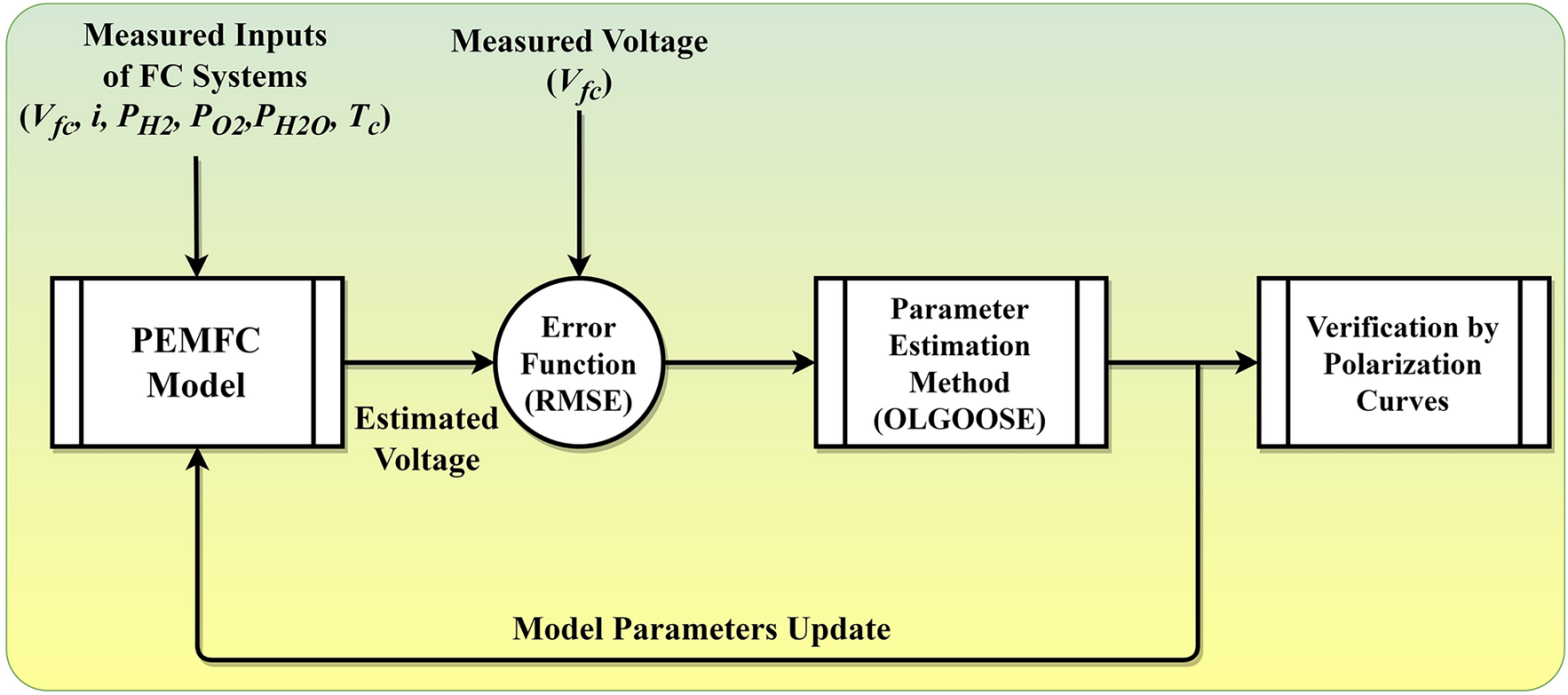
Block diagram representation of the proposed strategy.

Estimating these parameters is complex but crucial for operating the FC at optimal conditions and minimizing losses. The estimation of the parameter for a low-power, multiple-output, multiple-input electrochemical PEMFC structure has been conducted using current interruption tests and system identification approaches. Advanced algorithms have proven capable of deriving more accurate values than simpler methods. Beyond parametric coefficients, several critical factors impact the operation of FC systems significantly. These include reactant stoichiometry, stack current, stack temperature, humidity, and reactant pressure. Temperature plays a dominant role in fuel cell performance, while fuel pressure and flow rate have a lesser impact. In contrast, the airflow rate and air pressure, due to their negligible effect, are not considered for control purposes. Other influential factors encompass elements within the electrochemical and fluidic domains, such as thickness, porosity, gas diffusion coefficients between water and hydrogen and water and oxygen, respectively, gas diffusion layer tortuosity, in the fluidic domain. In the electrochemical domain, factors like symmetry, catalyst layer sectional area, exponential parameters of exchange current density, and scale factor are crucial. Various optimization methods have been utilized to determine these unknown FC parameters. Research has shown that while validating parameters obtained through optimization methods with empirical data, there is invariably some error. This parameter estimation process is depicted in Fig. 3.

A primary objective is to minimize the error between the experimental and estimated data, commonly using the fitness function of minimizing the Sum of Squared Errors (SSE), calculated from the differences between estimated data and experimental data collected at $N$ data samples. The objective is to minimize the SSE, and the error function is presented in Eq. (19).

19 $${\text{SSE}}={\sum }_{i=1}^{N}{\left({V}_{{\text{sim}},i}-{V}_{{\text{exp}},i}\right)}^{2}$$

where ${V}_{{\text{sim}},i}$ and ${V}_{{\text{exp}},i}$ are the simulated and experimental voltages at point $i$, respectively. Minimizing the difference between predicted and actual data is crucial for enhancing the reliability and efficiency of PEMFC systems. The Root Mean Square Error (RMSE) is used to evaluate the accuracy of model predictions against observed data in the parameter estimation process. The RMSE is derived from the SSE, and Eq. (20) presents the expression for RMSE.

20 $${\text{RMSE}}=\sqrt{\frac{\text{SSE}}{N}}$$

The optimization variables in the PEMFC model consist of seven unknown parameters, as presented in Eq. (21), and each parameter is subject to the constraints, as presented in Eq. (22).

21 $$x=[{\xi }_{1},{\xi }_{2},{\xi }_{3},{\xi }_{4},\lambda ,b,{R}_{c}]$$

22 $$\begin{array}{c}{\xi }_{i}^{min}\le {\xi }_{i}\le {\xi }_{i}^{max}\\ {\lambda }^{min}\le \lambda \le {\lambda }^{max}\\ {b}^{min}\le b\le {b}^{max}\\ {{R}_{C}}^{min}\le {R}_{C}\le {{R}_{C}}^{max}\end{array}$$

The unknown parameters are essential for the accurate modelling of the PEMFC’s behaviour and for enhancing its performance.

## Orthogonal learning based GOOSE algorithm

This section of the paper discusses the basic concepts of the GOOSE algorithm and the formulation of the proposed OLGOOSE algorithm.

### GOOSE algorithm

The GOOSE algorithm is a metaheuristic optimization algorithm inspired by the behaviours of geese during foraging and rest periods^87^. Initially, the GOOSE algorithm populates an array, denoted as the $X$ matrix, representing the geese's positions. Once populated, the algorithm repositions any search agents that stray outside the predefined search space. Each agent's fitness is evaluated in every iteration using standardized benchmark functions. The algorithm assesses and compares the fitness of each agent (each row in the $X$ matrix) against all others to identify the best fitness score and position, referred to as $BestFitness$ and $BestX$, respectively. To balance exploration and exploitation, a random variable named $"b"$ is utilized, and it commands the strategy, i.e., with a 50% chance, the algorithm chooses between exploring new areas or exploiting to refine the search. The distribution of phases is evenly managed across the iterations through a conditional statement. Additionally, several auxiliary variables, such as $"a"$, $"b",$ and $"c"$, are introduced to facilitate the decision-making process. These variables are generated randomly within the range of 0 to 1. A specific condition checks if $"c"$ exceeds 0.17; if it does, it is reset to 0.17 to maintain a controlled variability in the algorithm's behaviour. The variable $"a"$ is crucial in determining the position update.

In the exploitation phase of the GOOSE algorithm, a key requirement is to ensure group safeguarding, as discussed earlier. To achieve this, the algorithm randomly determines the stone weight supported by the goose, which ranges between 5 and 25, as per Eq. (23).

23 $$S\_{W}_{it}=randi([\text{5,25}],\text{1,1})$$

The algorithm calculates the time $T\_o\_A\_Oit$, which represents the duration needed for the stone to reach the Earth, randomly chosen between 1 and 0. In the subsequent formula, it is possible to calculate the overall period it takes for the sound to spread and influence each geese in the herd across all iterations. As detailed in Eq. (24), this total time is divided by the number of dimensions. The average time is then obtained by halving the total time, as outlined in Eq. (25).

24 $$T\_T=\frac{\sum (T\_o\_{A}_{it})}{dim}$$

25 $$T\_A=\frac{T\_T}{2}$$

As discussed earlier, the random variable ‘$b$’ is used to allocate the phases of exploration and exploitation. The value of ‘$a$’ is randomly chosen from between 0 and 1. If ‘$a$’ is greater than 0.2 and the weight ‘$S\_Wit$’ is 12 or more, Eq. (26) is applied where ‘$T\_o\_A\_Oit$’ is multiplied by the square root of ‘$S\_Wit$’ divided by 9.81 m/s^2^, the standard acceleration due to gravity.

26 $$F\_F\_S=T\_o\_A\_{O}_{it}*\frac{\sqrt[2]{S\_{W}_{it}}}{9.81}$$

Equation (27) calculates the distance sound travels, $D\_S\_Tit$, by multiplying the speed of sound in air, $S\_S$, which is 343.2 m per second, by the time it takes for the sound to travel, $T\_o\_A\_Sit$.

27 $$D\_S\_{T}_{it}=S\_S*T\_o\_A\_{S}_{it}$$

In this step, it is possible to calculate $D\_Git$, the distance between another goose at rest or feeding and a guard goose. Equation (28) determines this distance by taking half of the sound travel distance $D\_S\_{T}_{it}$.

28 $$D\_{G}_{it}=0.5*D\_S\_{T}_{it}$$

To update a position within the population, specifically to awaken an individual in the flock, it needs to determine ${BestX}_{it}$ as outlined in Eq. (29), and it combines the falling object $F\_F\_S$ with the product of the goose's distance $D\_{G}_{it}$ and the square of the average time $T\_A$.

29 $${X}_{(it+1)}=F\_F\_S+D\_{G}_{it}*T\_{A}^{2}$$

Conversely, if both stone weight $S\_{W}_{it}$ and $a$ are less than 12, and $a$ is less than or equal to 0.2, the new position $X$ is calculated as described in Eq. (30). To compute the falling object speed $F\_F\_S$, multiply the time $T\_o\_A\_{O}_{it}$, it takes for the object to arrive by the stone weight $S\_{W}_{it}$ divided by gravity. Furthermore, the distances of sound travel $D\_S\_{T}_{it}$ and the goose $D\_{G}_{it}$ are calculated using the earlier Eqs. (27) and (28).

30 $$F\_F\_S=T\_o\_A\_{O}_{it}*\frac{S\_{W}_{it}}{9.81}$$

Alternatively, a new position $X$ is calculated using the formula outlined in Eq. (31), where parameters such as the falling object speed, goose distance, mean time, and coefficient $c$ are sequentially multiplied. In the exploitation phase, Eqs. (28) and (30) are used to compute a new $X$. The choice between these equations is determined by the values of variables $a$ and $W\_Sit$.

31 $${X}_{(it+1)}=F\_F\_S*D\_{G}_{it}*T\_{A}^{2}*c$$

In the exploitation stage, the goose wakes randomly in response to the best position exposed so far, either to control its wake-up or to protect the individual in the flock. In addition, it is necessary to ensure that if the minimum time $M\_T$ exceeds the total time $T\_T$, $M\_T$ is then set to equal $T\_T$. The variable $alpha$, which ranges from 2 to 0, decreases significantly with each iteration. This reduction is captured in Eq. (32), which is employed to refine the positioning of a new $X$ within the search space.

32 $$alpha=\left(2-\left(\frac{loop}{\frac{Ma{x}_{it}}{2}}\right)\right)$$

In this context, $Ma{x}_{it}$ represents the maximum number of iterations allowable. Calculating the parameters $M\_T$ (minimum time) and $alpha$ is essential to steer the search phase towards what is likely the optimal solution. It is significant to enable the goose to stochastically explore the positions of other populations in the search location, which is achieved by means of $randn(1, dim)$. The variables $M\_T$ and $alpha$ are important in enhancing the search capabilities of the GOOSE algorithm. In Eq. (33), a random number is multiplied by the minimum of time and $alpha$, and this product is subsequently added to the optimal location found in the search location, facilitating effective exploration and exploitation.

33 $${X}_{(it+1)}=randn(1,dim)*(M\_T*alpha)+Best\_pos$$

where $dim$ denotes the problem dimensions, and $Best\_pos$ denotes the top position found so far in the search area. The pseudocode is provided in Algorithm 1.

**Figure:**
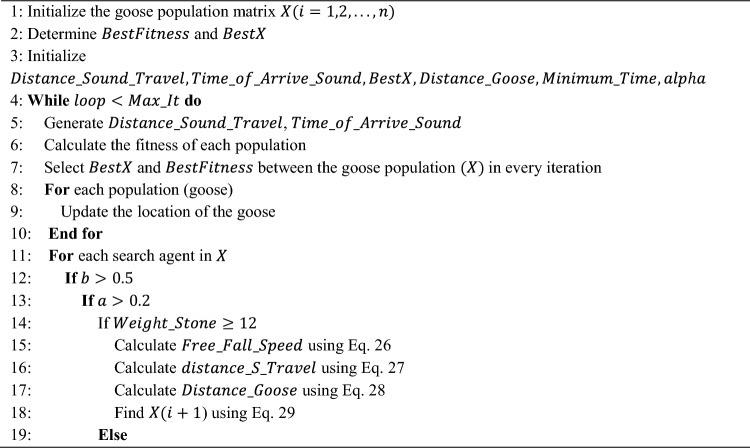
Algorithm 1: Pseudocode of the GOOSE algorithm

### Orthogonal learning

Orthogonal Learning (OL) is a concept derived from the mathematical property of orthogonality, where two vectors are orthogonal if their dot product is zero, indicating that they are perpendicular to each other^88,89^. This principle can be applied to optimization algorithms to enhance their search strategies by ensuring diversity in the search directions. In OL, the search agents (or solutions) are encouraged to explore the search space in directions that are orthogonal to each other. This means that each agent explores a fundamentally different aspect or dimension of the problem space, reducing redundancy in the search process and covering more areas more efficiently. By utilizing orthogonal vectors, the algorithm can effectively escape local optima. Each orthogonal vector points in a direction that is not influenced by the others, ensuring that the agents do not cluster around local optima and instead explore more globally^90,91^. The OL helps in balancing exploration and exploitation. As the search progresses, the degree of orthogonality can be adjusted to focus more on exploitation, particularly as the algorithm converges towards potential solutions. The OL method is adaptable to various types of optimization problems because it does not depend heavily on the gradient of the problem space, making it suitable for non-differentiable, noisy, or highly complex landscapes.

The OL in optimization algorithms involves the use of vectors that are mutually orthogonal to each other, thus ensuring that search agents explore the search space along independent directions^92^. Two vectors $u$ and $v$ in an $n$-dimensional space are orthogonal if their dot product is zero:

34 $$u\cdot v=0$$

For a set of vectors to be mutually orthogonal, every pair of different vectors in the set must satisfy this condition. In practice, this can be achieved through processes such as Gram-Schmidt orthogonalization or by using predefined orthogonal matrices like Hadamard matrices in cases where dimensions allow. One straightforward method for generating orthogonal vectors in the context of an optimization algorithm is to use the QR decomposition of a randomly generated matrix^93^. Suppose $A$ is a $n \times n$ matrix with randomly generated entries. The QR decomposition of $A$ is:

35 $$A=QR$$

where $Q$ is an orthogonal matrix (the columns are orthogonal unit vectors), and $R$ is an upper triangular matrix. The columns of $Q$ can be used as directions for orthogonal exploration.

### Proposed OLGOOSE algorithm

In the context of the GOOSE algorithm, which is inspired by the natural behaviour of geese, orthogonal learning can significantly enhance its performance by integrating the following modifications and improvements: (i) During the initialization phase, the OL method can be applied to generate initial positions of the geese (search agents) so that they are spread out over the search space in a manner that minimizes overlap and redundancy; (ii) In each iteration, instead of moving solely based on the best solution found or random perturbations, the geese can also move in directions that are orthogonal to the direction of the current best solution; (iii) As the algorithm progresses, the extent of orthogonality in the moves can be dynamically adjusted. Early in the search process, high orthogonality can be beneficial for broad exploration, while later in the process, reducing orthogonality can help in fine-tuning the solutions by focusing more on exploitation near the current best areas; (iv) By integrating OL, the GOOSE algorithm can achieve faster convergence rates and better global optima discovery. The orthogonal directions ensure that the search is not trapped in local optima and that the solution space is thoroughly explored.

The initialization of agents (geese) can be modelled using the orthogonal matrix $Q$. For a set of initial agents $X$ in a $n$-dimensional space:

36 $$X=Q\times D$$

where ${\varvec{D}}$ is a diagonal matrix whose diagonal elements are scaled according to the problem's bounds (i.e., the search space limits). During the iterative process, the algorithm can adjust each agent's position using orthogonal directions derived from the best current position ${X}_{\text{bs}}$:

37 $$X_{{i,{\text{new}}}} = X_{i} + \alpha \cdot Q_{i}$$

where $\alpha$ is a step size, and ${{\varvec{Q}}}_{{\varvec{i}}}$ is the $i$th orthogonal vector influencing the direction of the $i$th agent. As the search progresses, the degree of orthogonality can be controlled by a parameter $\upbeta ,$ which modulates the influence of orthogonal directions based on the phase of the optimization:

38 $$X_{{i,{\text{new}}}} = X_{i} + {\upbeta }\left( t \right) \cdot \alpha \cdot Q_{i}$$

where $\upbeta \left(t\right)$ decreases as the number of iterations increases, reducing the influence of orthogonal directions to allow more localized search near the end of the algorithm run. By initializing and guiding search agents in orthogonal directions, the algorithm covers the search space more comprehensively, reducing the risk of missing global optima. Orthogonal steps help maintain diversity in the population of agents, preventing them from clustering around local optima too early in the search process. With dynamic adjustment of the orthogonality parameter, the algorithm effectively transitions from broad exploration to intensive exploitation, optimizing performance over iterations. This orthogonal modelling enhances the robustness and effectiveness of the GOOSE algorithm, particularly in complex, high-dimensional search spaces where traditional methods may struggle with coverage and convergence. The pseudocode of the proposed OLGOOSE algorithm is shown in Algorithm 2.

**Figure:**
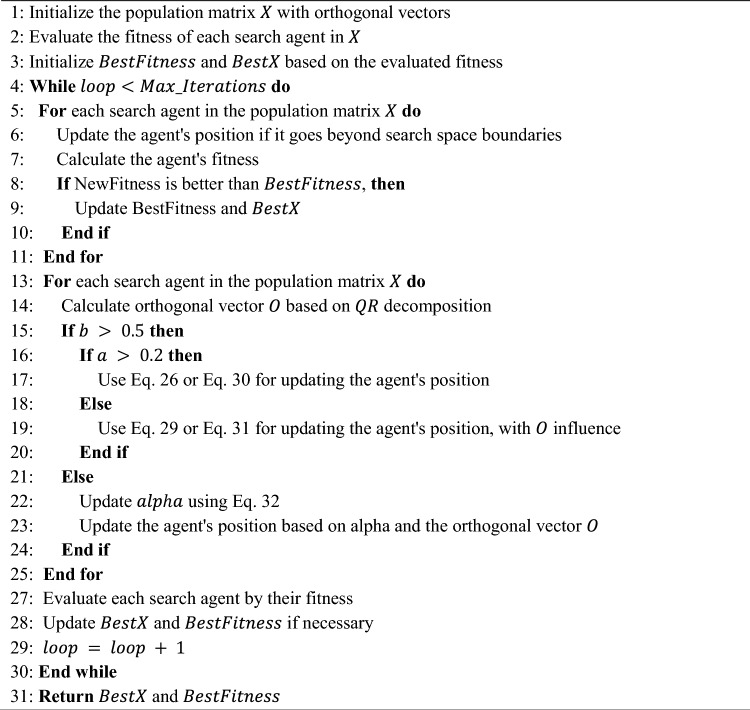
Algorithm 2: Pseudocode of the proposed OLGOOSE algorithm

### Complexity of the OLGOOSE Algorithm

The orthogonal initialization of the population matrix $X$ using QR decomposition has a time complexity of $O\left({n}^{3}\right)$ for a matrix of size $n\times n$. However, since the matrix size is typically $m\times n$ where $m$ is the number of population and $n$ is the problem dimension, the initialization complexity would be $O\left(m\cdot {n}^{2}\right),$ if we assume full orthogonalization for simplicity. Each agent’s fitness is evaluated once per iteration, which gives us $O\left(m\right)$ per iteration, assuming the fitness evaluation function has a constant time complexity. If the fitness function has a complexity of $O\left(f\right)$, then this step is $O\left(m\cdot f\right)$. Updating the position of each agent includes computing orthogonal vectors and potentially performing the QR decomposition in each iteration, which has a time complexity of $O\left(m\cdot {n}^{2}\right)$. Combining all these gives us the overall time complexity of the Orthogonal Learning based GOOSE algorithm: $O\left(k\cdot \left(m\cdot {n}^{2}+m\cdot f\right)\right)$, $k$ denotes the number of runs.

The space complexity is calculated by the amount of memory needed to store data structures at any point in the algorithm: (i) Stores the position of each agent, requiring $O\left(m\cdot n\right)$ space; (ii) Storing the best position and fitness requires $O\left(n\right)$ and $O\left(1\right)$ space, respectively; (iii) During orthogonal learning, orthogonal vectors can be stored in a matrix of size $m\times n$, which is $O\left(m\cdot n\right)$; (iv) Variables like ${\text{BestFitness}}$, ${\text{BestX}}$, $distances$, $times$, etc., add a marginal additional space requirement, which is typically $O\left(m\right)$ or $O\left(n\right)$, depending on whether they store per-agent or per-dimension data. Therefore, the space complexity is $O\left(m\cdot n\right)$.

## Results and discussions

The process of identifying the parameters was conducted using MATLAB software. Initially, the parameters in the model were assigned random values within ranges, as presented in Table 1. Data from the FC was then transferred to the identification program for analysis, where it was aligned and compared with the outputs from the model. The differences between the actual data and the model outputs were encapsulated within the objective function, as detailed in Eq. (17), with parameter adjustments made iteratively. For the estimation of the unknown parameters, the proposed OLGOOSE strategy was applied to three PEM fuel cells: the NedStackPS6, SR-12, and BCS 500 W models. To validate the efficacy of the OLGOOSE strategy, its performance was evaluated against several other optimization algorithms, including the GOOSE, Gradient-Based Optimizer (GBO)^58^, Multi-Learning Reptile Search Algorithm (MLRSA)^45^, the Subtraction-Average-Based Optimizer (SABO)^94^, the Energy Valley Optimizer (EVO)^95^, the Black Widow Optimization Algorithm (BWOA)^50^, and the Marine Predator Algorithm (MPA)^69^. The operating conditions, parameters, and datasets for these specific PEMFC stacks were sourced from^12,77^. Details regarding the specifications of the PEMFCs under study are presented in Table 2. Notably, the cathode was supplied with air for the BCS 500W and SR-12 Modular types, whereas the NedStackPS6 type was supplied with pure oxygen. This distinction is crucial as it impacts the fractional pressure of oxygen (${P}_{{O}_{2}}$) and the overall performance of the FCs.

**Table 1:** Parameter limits for the FC model.

Parameters	${\xi }_{1}$	${\xi }_{2}$	${\xi }_{3}$	${\xi }_{4}$	$b$	$\lambda$	${R}_{c}$
Lower bounds	− 1.19969	$0.001$	$3.6\times {10}^{-5}$	$-2.6\times {10}^{-4}$	0.136	10	$1\times {10}^{-4}$
Upper bounds	− 0.08532	$0.005$	$9.8\times {10}^{-5}$	$-9.54\times {10}^{-5}$	0.5	24	$8\times {10}^{-4}$

**Table 2:** Specifications of the PEMFC cells and stacks.

Type	BCS 500W	SR-12 Modular	NedStackPS6
$N$ (cells)	32	48	65
$A$ (cm^2^)	64	62.5	240
$l \left(\upmu m\right)$	178	25	178
${PH}_{2 }$($\text{atm}$)	1	1.47628	1
${PO}_{2}$($\text{atm}$)	1	0.2095	1
${i}_{max} \left(\frac{\text{A}}{{\text{cm}}^{2}}\right)$	0.469	0.672	5
$T$(K)	333	323	343
Cathode supply	Air	Air	Oxygen

To ensure a balanced comparison, the number of populations and the number of iterations were standardized across all optimization algorithms, set at 40 and 1000, respectively. Throughout the optimization process, the objective function used was the RMSE between the measured voltage data and the calculated voltage outputs from the selected FC model. The goal was to minimize this RMSE. The unidentified parameters of the PEMFC functioned as the decision vectors within the optimization framework. The specific upper and lower bounds for these PEMFC variables are detailed in Table 1.

### Results for all FC models

Table 3 showcases the optimal parameters obtained for various fuel cell models when subjected to different algorithms. Due to the inherently random characteristics of algorithms, the outcomes they produce can vary from one execution to another. This variability stems from the algorithms' design, which incorporates randomness to escape local optima and explore the search space extensively. To account for the stochastic behaviour of these algorithms and to ensure a robust evaluation, multiple executions are necessary, i.e., 30 individual executions. This approach helps in assessing the performance consistency of each algorithm across runs. By averaging results from several iterations, it is possible to mitigate the influence of the outlier results, providing a more accurate reflection of the algorithm's capability to identify optimal parameters reliably. For the performance comparison, error metrics are considered. Error metrics are quantitative measures used to assess the accuracy of algorithms in predicting or fitting data. A brief introduction to the error metrics is discussed as follows.

**Table 3:** Estimated parameters of PEMFC by all algorithms.

Parameters	OLGOOSE	GOOSE	OBGBO	MLRSA	SABO	EVO	RLBWOA	OBMPA
BCS 500W								
ξ1	− 1.09997	− 0.24035	− 0.49072	− 0.44035	− 0.75393	− 0.58400	− 0.65772	− 0.95348
ξ2	0.00319	0.00100	0.00100	0.00121	0.00249	0.00130	0.00201	0.00266
ξ3	0.00006	0.00010	0.00004	0.00006	0.00009	0.00004	0.00007	0.00006
ξ4	− 0.00019	− 0.00012	− 0.00019	− 0.00019	− 0.00019	− 0.00019	− 0.00019	− 0.00019
Λ	23.99857	14.25850	20.87724	19.18349	22.08166	22.23056	20.10355	20.87724
b (V)	0.01630	0.02240	0.01613	0.01486	0.01497	0.01636	0.01464	0.01613
*R*_*c*_ (Ω)	0.00040	0.00010	0.00010	0.00011	0.00049	0.00021	0.00020	0.00010
MAE	1.291E−02	4.056E−01	1.309E−02	1.580E−02	1.216E−01	1.308E−02	1.800E−01	1.291E−02
SSE	1.170E−02	5.629E+00	1.177E−02	1.366E−02	5.012E−01	1.216E−02	2.967E+00	1.170E−02
RMSE	0.02549	0.21290	0.02549	0.02562	0.03542	0.02549	0.02667	0.02549
NedStackPS6								
ξ1	− 1.03626	− 0.30462	− 0.36150	− 0.27798	− 0.30166	− 0.30484	− 0.21725	− 0.58890
ξ2	2.93E−03	7.99E−04	1.09E−03	8.11E−04	7.99E−04	8.00E−04	7.99E−04	1.91E−03
ξ3	3.60E−05	3.60E−05	4.48E−05	4.24E−05	3.60E−05	3.60E−05	5.43E−05	5.62E−05
ξ4	− 9.54E−05	− 9.66E−05	− 9.66E−05	− 9.66E−05	− 9.66E−05	− 9.66E−05	− 9.66E−05	− 9.66E−05
Λ	13.02226	21.56335	13.02226	13.28265	20.37303	13.02226	13.02226	13.02226
b (V)	0.01360	0.11653	0.01360	0.01905	0.08862	0.01360	0.01360	0.01360
*R*_*c*_ (Ω)	0.00010	0.00010	0.00010	0.00010	0.00020	0.00010	0.00010	0.00010
MAE	0.20671	0.25614	0.20725	0.21532	0.21991	0.20573	0.20563	0.20563
SSE	2.10422	3.34063	2.11666	2.28959	2.35557	2.07966	2.07965	2.07933
RMSE	0.26777	0.27582	0.26777	0.26882	0.27146	0.26777	0.26777	0.26777
SR− 12								
ξ1	− 0.22942	− 0.37122	− 1.13148	− 0.60555	− 0.72124	− 0.37027	− 1.19969	− 0.48195
ξ2	1.077E−03	8.000E−04	4.112E−03	1.660E−03	2.220E−03	8.000E−04	3.404E−03	1.488E−03
ξ3	8.230E−05	3.600E−05	9.797E−05	4.474E−05	5.687E−05	3.600E−05	3.847E−05	5.833E−05
ξ4	− 9.540E−05	− 9.540E−05	− 9.540E−05	− 9.540E−05	− 1.019E−04	− 9.540E−05	− 1.019E−04	− 9.540E−05
λ	23.50649	22.14761	10.00015	12.96875	10.56164	15.10609	23.92702	23.50650
b (V)	0.17391	0.17351	0.16945	0.17347	0.16786	0.17029	0.18459	0.17391
*R*_*c*_ (Ω)	0.00080	0.00080	0.00054	0.00055	0.00052	0.00080	0.00011	0.00080
MAE	0.19998	0.20514	0.19999	0.20598	0.20587	0.19994	0.20738	0.19998
SSE	1.33104	1.39692	1.33105	1.36410	1.37992	1.33404	1.39320	1.33104
RMSE	0.27193	0.27198	0.27193	0.27292	0.27314	0.27193	0.27232	0.27193

Mean absolute error (MAE): MAE measures the average magnitude of errors in a set of predictions without considering their direction. It is the mean of the absolute values of each error.

39 $$MAE=\frac{1}{N}{\sum }_{i=1}^{N}\left|{V}_{{\text{sim}},i}-{V}_{{\text{exp}},i}\right|$$

where $N$ is the number of samples, ${V}_{{\text{sim}},i}$ is the estimated value, and ${V}_{{\text{exp}},i}$ is the experimented value.

Sum of Squared Errors (SSE): SSE calculates the total sum of squared differences between the predicted and actual values. It emphasizes larger errors due to squaring.

40 $$SSE={\sum }_{i=1}^{n}{\left({V}_{{\text{sim}},i}-{V}_{{\text{exp}},i}\right)}^{2}$$

In Table 3, several algorithms are compared, including OLGOOSE, across multiple datasets based on different performance parameters. OLGOOSE shows distinct advantages in optimization, with the data indicating its superior efficacy. OLGOOSE stands out primarily in its optimization precision, achieving the best or near-best scores in most parameters. For instance, in the BCS 500W dataset, the proposed algorithm hits the closest value to the optimum for the parameter ${\upxi }_{1}$, indicating its effectiveness in fine-tuning the distinctions of the dataset. The OLGOOSE consistently maintains low error metrics for all case studies, representing its capability to converge to the optimal solution with minimal deviation. The low values of $b$ and $Rc$ recommend that OLGOOSE controls voltage variations and resistance, and it is crucial for stable and accurate estimation. Furthermore, the high $\uplambda$ values indicate the high solution’s quality. OLGOOSE’s consistent performance across all the estimated parameters suggests that the proposed OLGOOSE not only surpasses in highlighting the exact optimal points but also reliably maintains the performance, showing flexibility and robustness. Through the investigation of the estimated parameters and error metrics, OLGOOSE’s position as a reliable algorithm is likely to provide better outcomes when handling complex optimization problems. The improved results obtained by OLGOOSE, as compared to other algorithms like GOOSE, OBGBO, MLRSA, SABO, EVO, RLBWOA, and OBMPA, demonstrate the impact of orthogonal learning on the algorithm’s performance, offering a significant enhancement over traditional methods.

The data shown in Table 4 illustrates that the OLGOOSE algorithm shows notable consistency and efficiency when compared to other algorithms across various metrics and datasets. OLGOOSE consistently maintains a competitive edge in BCS 500W, NedStackPS6, and SR-12. OLGOOSE has remarkable accuracy in its average values, closely aligning with the minimal error rates in all the datasets. The standard deviation (STD) of this is remarkably low, often reaching the lower limits of accuracy, suggesting its constant and exact performance across multiple runs. The low STD values highlight the stability of OLGOOSE. The other statistical parameters, such as minimum (Min) and maximum (Max) values, suggest that OLGOOSE not only demonstrates strong performance on average but also effectively avoids any prominent outliers that could obstruct optimization in practical circumstances.

**Table 4:** Obtained statistical metrics by all algorithms.

Metric	OLGOOSE	GOOSE	OBGBO	MLRSA	SABO	EVO	RLBWOA	OBMPA
BCS 500W								
Mean	**0.02549**	0.48062	0.02565	0.02874	0.09531	0.02631	0.03750	**0.02549**
STD	**1.270E−15**	1.435E−01	2.830E−04	2.973E−03	6.573E−02	1.062E−03	1.911E−02	1.360E−15
Min	**0.02549**	0.21290	**0.02549**	0.02562	0.03542	**0.02549**	0.02667	**0.02549**
Max	0.02549	0.66441	0.02641	0.03508	0.20435	0.02869	0.09079	0.02549
RT	1.909	**1.857**	3.303	2.802	2.985	2.055	2.792	5.731
FRT	**1.300**	8	3	5	6.900	3.900	5.900	2
NedStackPS6								
Mean	0.26856	0.30093	**0.26777**	0.27782	0.28788	0.26791	0.32581	**0.26777**
STD	2.50E−03	7.79E−02	**3.09E−13**	7.36E−03	1.21E−02	4.07E−04	1.80E−01	5.12E−13
Min	**0.26777**	0.27582	**0.26777**	0.26882	0.27146	**0.26777**	**0.26777**	**0.26777**
Max	0.27566	0.52263	0.26777	0.29018	0.30933	0.26906	0.83743	0.26777
RT	3.570	**3.113**	4.769	5.041	4.215	5.256	5.025	8.183
FRT	3.700	6.800	**1.550**	6.400	7.400	3.950	3.650	2.550
SR-12								
Mean	**0.27193**	0.28843	0.27209	0.27467	0.28730	0.27248	0.27476	**0.27193**
STD	**1.302E−16**	3.777E−02	4.925E−04	1.990E−03	1.543E−02	6.248E−04	2.049E−03	4.367E−13
Min	**0.27193**	0.27198	**0.27193**	0.27292	0.27314	**0.27193**	0.27232	**0.27193**
Max	0.27193	0.39487	0.27349	0.27869	0.31854	0.27346	0.27952	0.27193
RT	1.992	**1.907**	3.425	2.860	3.048	3.089	4.854	5.830
FRT	**1.600**	6.100	2.900	6.100	7.500	4.100	5.900	1.800

Significant values are given in bold.

OLGOOSE demonstrates worthy outcomes in terms of runtime (RT), which quantifies the efficiency and speed of categorizing the initial acceptable solution. The proposed algorithm demonstrates less RT in multiple cases, suggesting a quick approach to reaching optimal solutions; however, the RT values are slightly higher compared to the original GOOSE algorithm. When considering Friedman’s ranking test (FRT), OLGOOSE’s rankings are outstanding, and the FRT metric is essential for the comparative performance of algorithms, providing a comprehensive assessment rather than focusing on individual instances. When compared with other algorithms, the proposed OLGOOSE demonstrates superior performance. The quality of the algorithm is validated by its low error metrics and top ranks in FRT values. OLGOOSE constantly exhibits its strength and expertise in executing activities with accuracy and effectiveness in many testing conditions. OLGOOSE is a powerful choice for improving complicated problems in various applications because of its fast convergence and dependable solutions. The results confirm that OLGOOSE is a well-optimized algorithm, consistently delivering better results than other algorithms in the comparison.

**Fig. 4:**
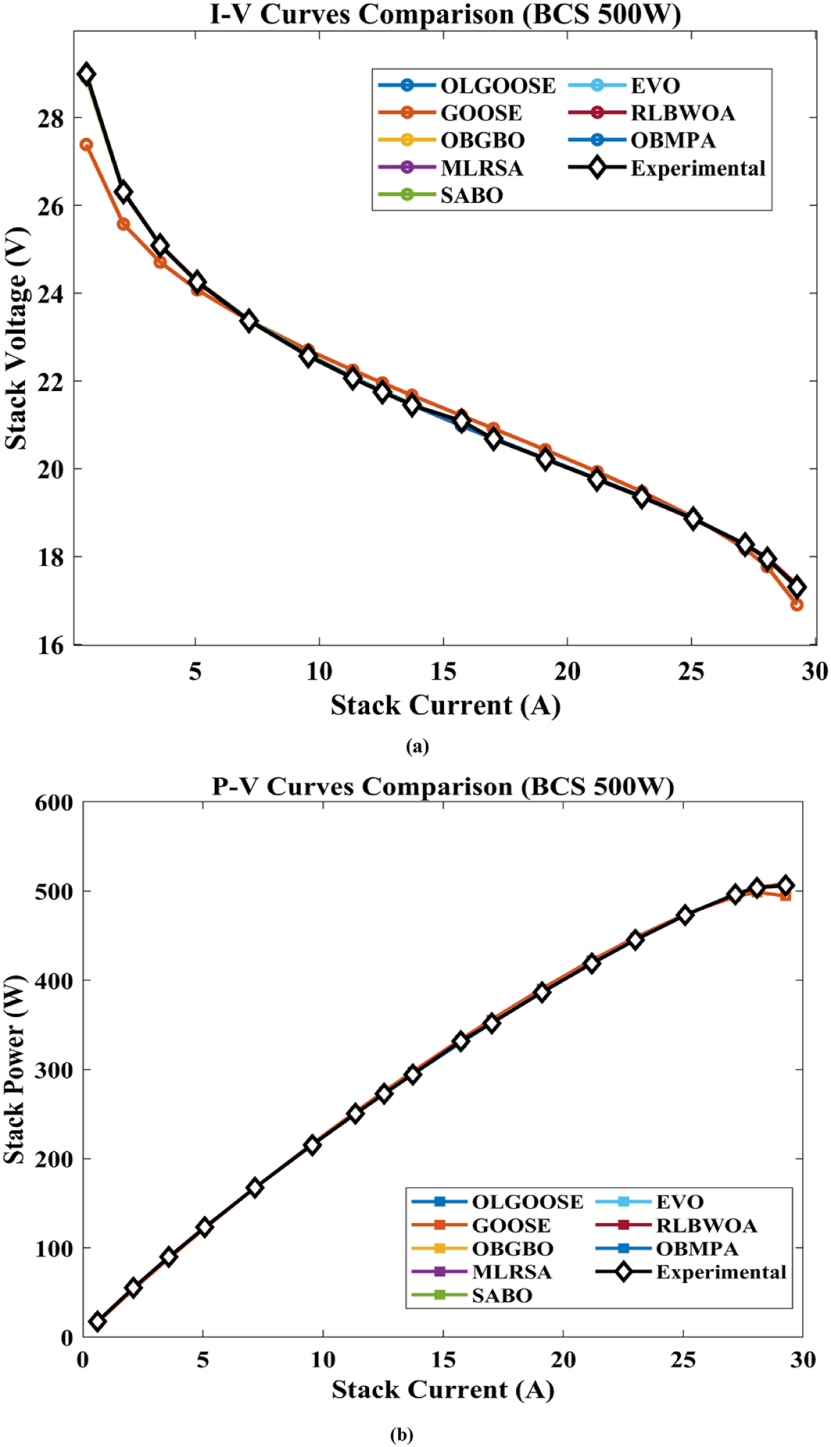
Characteristics of BCS 500W FC model; (**a**) I–V curves, (**b**) I–P curves.

A detailed analysis comparing the experimental values with the estimated values for the BCS 500 W fuel cell, obtained using the OLGOOSE algorithm, is illustrated in Fig. 4. This comparison shows a high degree of fit between the estimated voltages generated by the OLGOOSE algorithm and the actual experimental measurements, indicating strong model accuracy and effective parameter estimation.

**Fig. 5:**
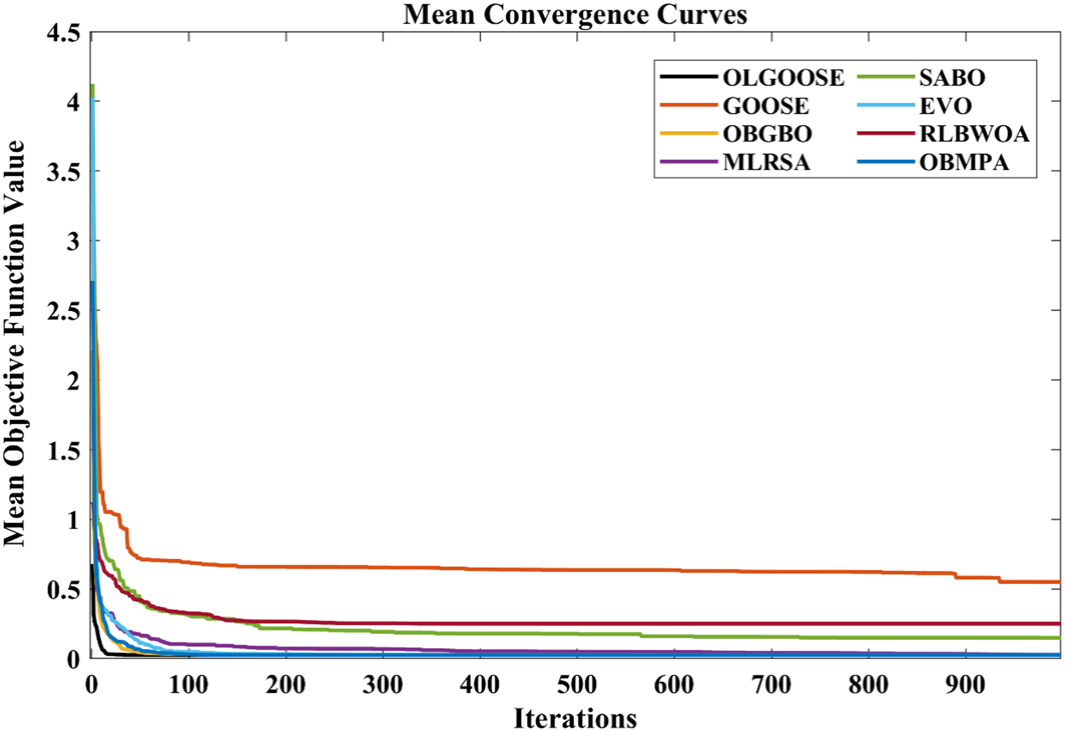
Convergence curves of all algorithms (BCS 500W FC model).

The quantitative evaluation of this fit, as detailed in Table 4, includes several error metrics: the MAE is reported at 1.291E−02, the SSE at 1.17E−02, and the RMSE at 0.02549. The low error metrics confirm the usefulness of the OLGOOSE algorithm in precisely determining the optimal settings for the BCS 500 W stack. Furthermore, Fig. 5 illustrates the changes in the fitness function during the process of determining the parameters for the BCS 500 W FC. The convergence curves for all the investigated algorithms are shown in Fig. 5, illustrating the efficiency and speed at which each algorithm converges towards the optimal solution. Figure 5 highlights the robustness and computational efficiency of OLGOOSE in accurately finding the optimal set of parameters. To summarize, the presented statistics and figures validate the assertion that the OLGOOSE approach is superior in precisely estimating parameters for the BCS 500 W stack.

**Fig. 6:**
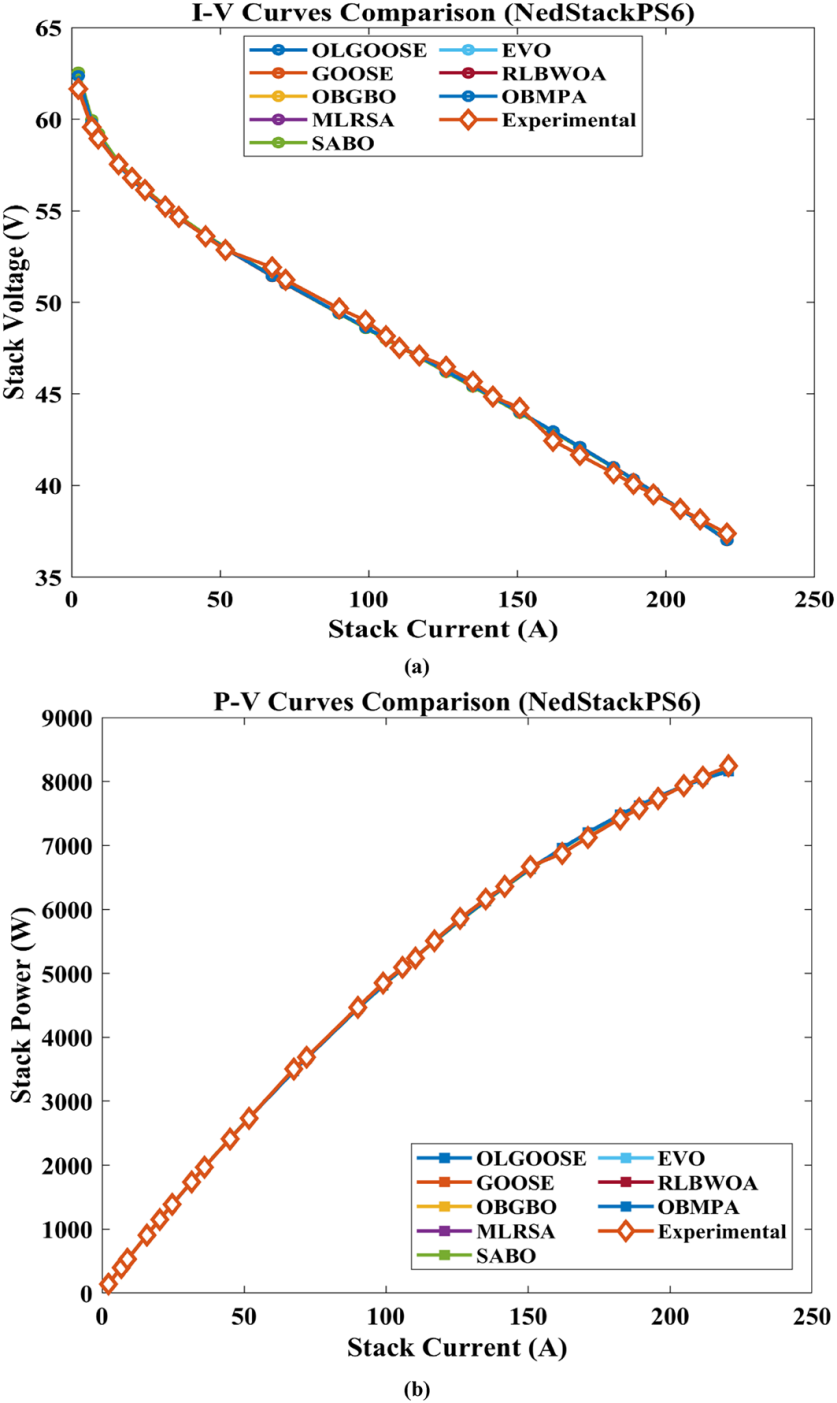
Characteristics of NedStackPS6 FC model; (**a**) I–V curves, (**b**) I–P curves.

**Fig. 7:**
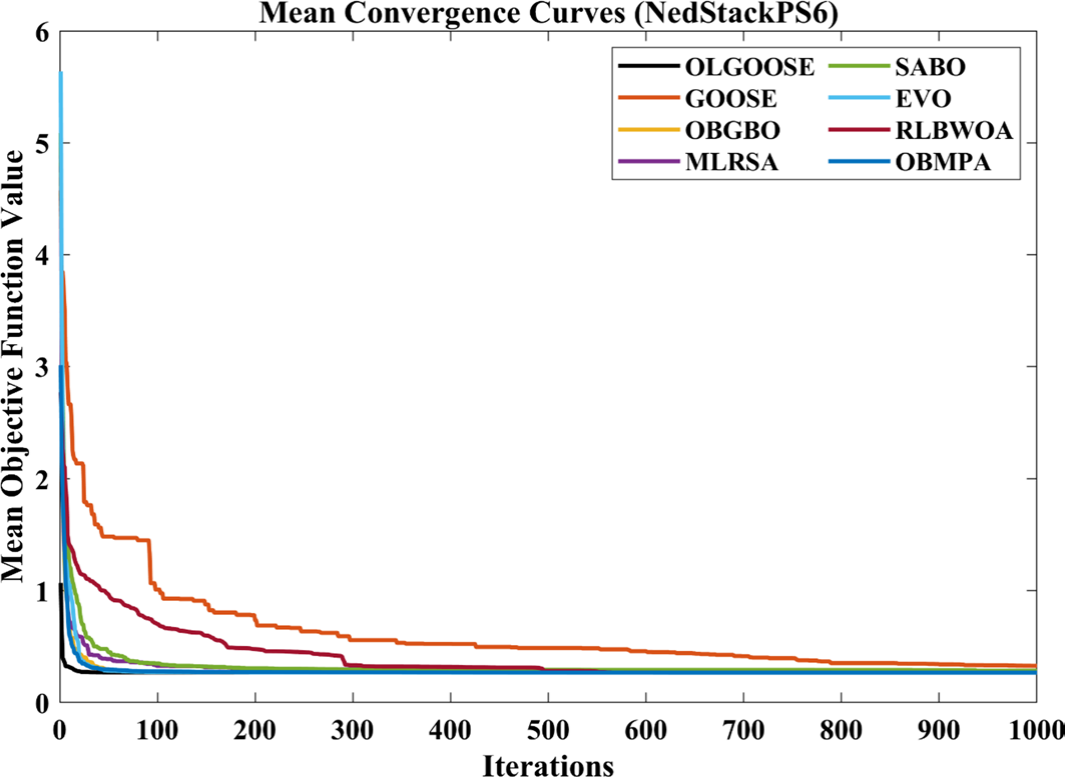
Convergence curves of all algorithms (NedStackPS6 FC model).

Figure 6 presents a comprehensive analysis of the measured voltage data and the simulated voltage values for the NedStackPS6 FC. Figure 6 demonstrates a strong correlation between the estimated voltages generated by OLGOOSE and the experimental findings, suggesting a successful alignment and efficient modelling by the proposed algorithm and all other selected algorithms. The effectiveness of the OLGOOSE algorithm in selecting the best parameters for the NedStackPS6 is further confirmed by the error metrics provided in Table 4. The MAE is measured at 0.20671, the SSE at 2.10422, and the RMSE at 0.26777. The low MAE, SSE, and RMSE indicate that the OLGOOSE method is capable of consistently and precisely determining the optimal parameters for the NedStackPS6 stack. As a result, the model’s predictions closely align with the actual data. Figure 7 displays the changes in the fitness function as the parameters are determined for the NedStackPS6 stack. Figure 7 displays convergence curves for all algorithms, demonstrating the evolution of each approach towards the best solution during the optimization process. Figure 7 illustrates the convergence behaviour, which may be used as a visual benchmark to evaluate the effectiveness of the OLGOOSE method in selecting the best parameter set compared to other algorithms.

**Fig. 8:**
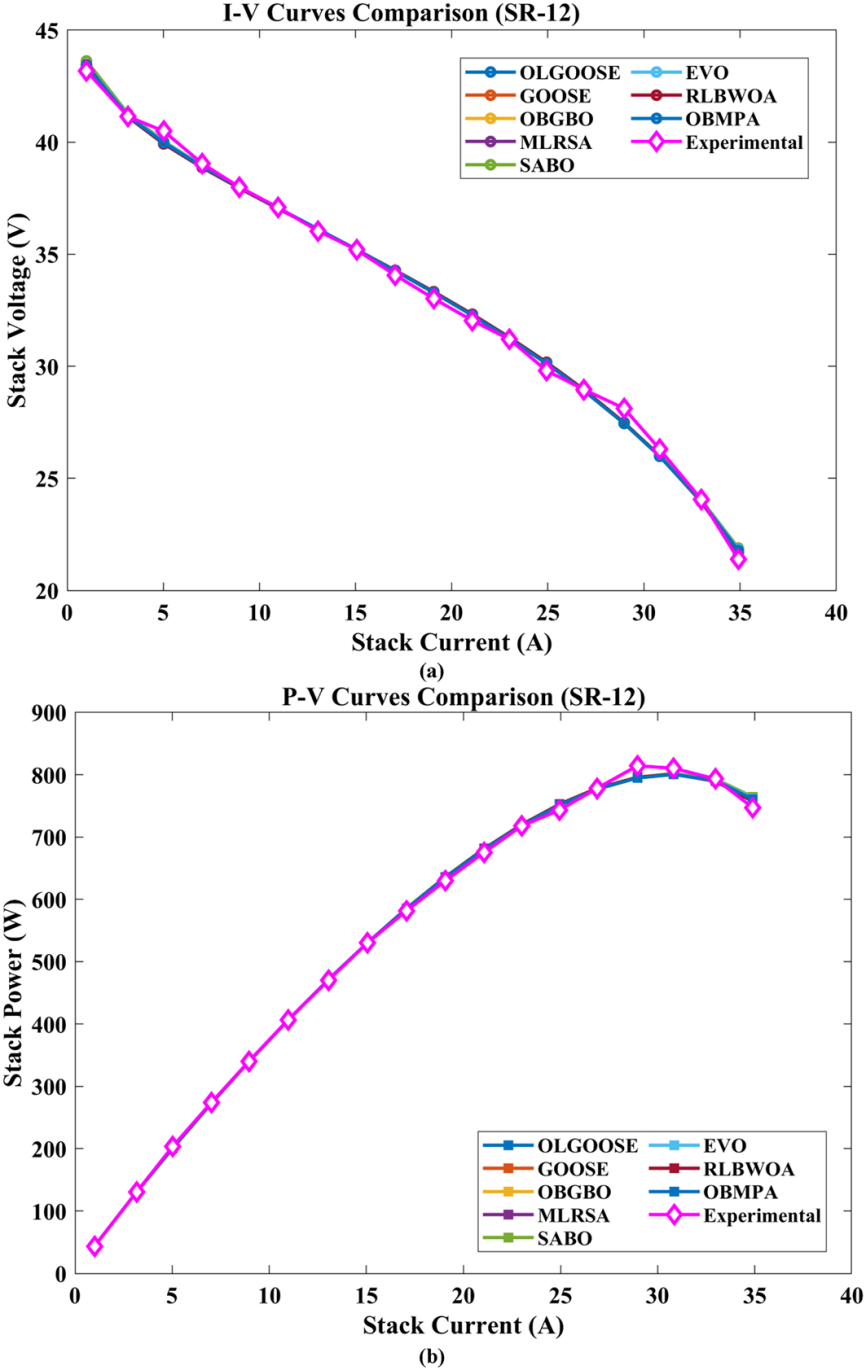
Characteristics of SR-12 FC model; (**a**) I–V curves, (**b**) I–P curves.

**Fig. 9:**
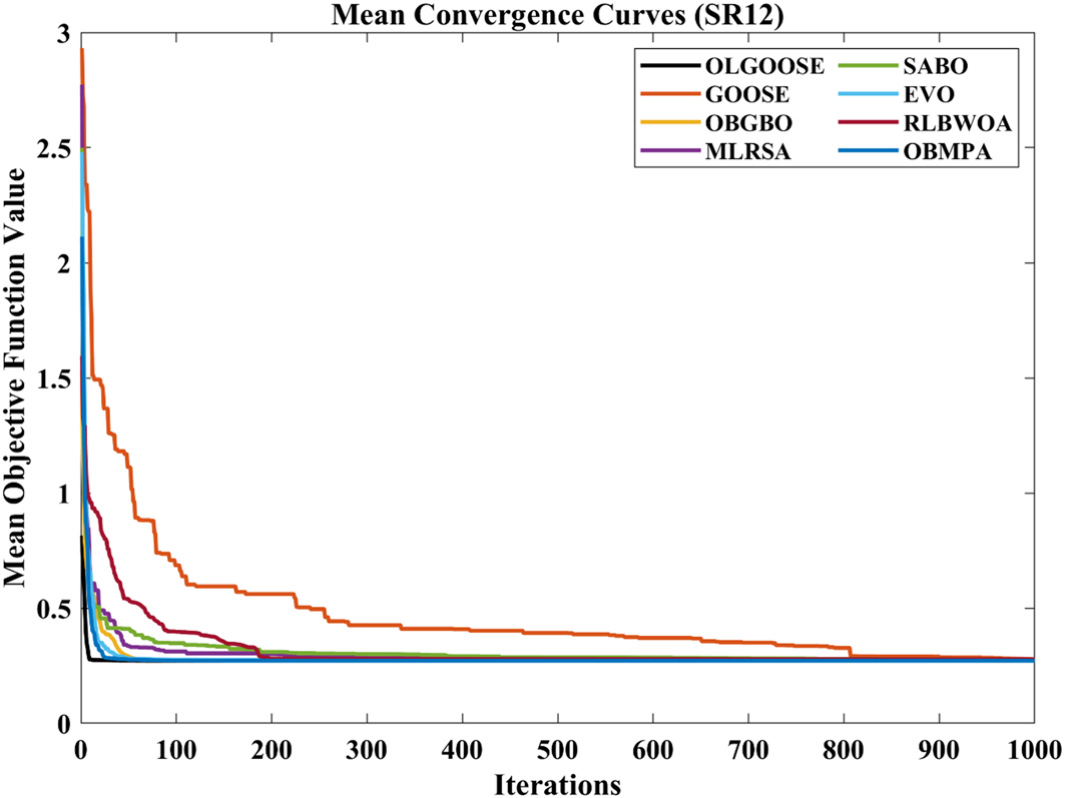
Convergence curves of all algorithms (SR-12 FC model).

Figure 8 provides an insightful comparison between the measured voltage data and the data estimates derived from the OLGOOSE algorithm for the SR-12 fuel cell model. The comparison discloses that the voltage values estimated by OLGOOSE closely align with the experimental measurements. The successful alignment of the actual data and predicted data indicates that the OLGOOSE algorithm is highly effective in modelling the behaviour of the SR-12 fuel cell under varied operational conditions. Further validation of the OLGOOSE algorithm’s efficacy comes from the detailed error metrics reported in Table 4. These include an MAE of 0.19998, SSE of 1.33104, and RMSE of 0.27193. Additionally, Fig. 9 illustrates the fitness function variations during the parameter optimization process for the SR-12 stack, including convergence curves for all algorithms compared. The convergence curves specifically highlight how quickly and smoothly the OLGOOSE algorithm approaches the optimal parameters, reflecting its computational efficiency and robustness in parameter optimization tasks.

**Fig. 10:**
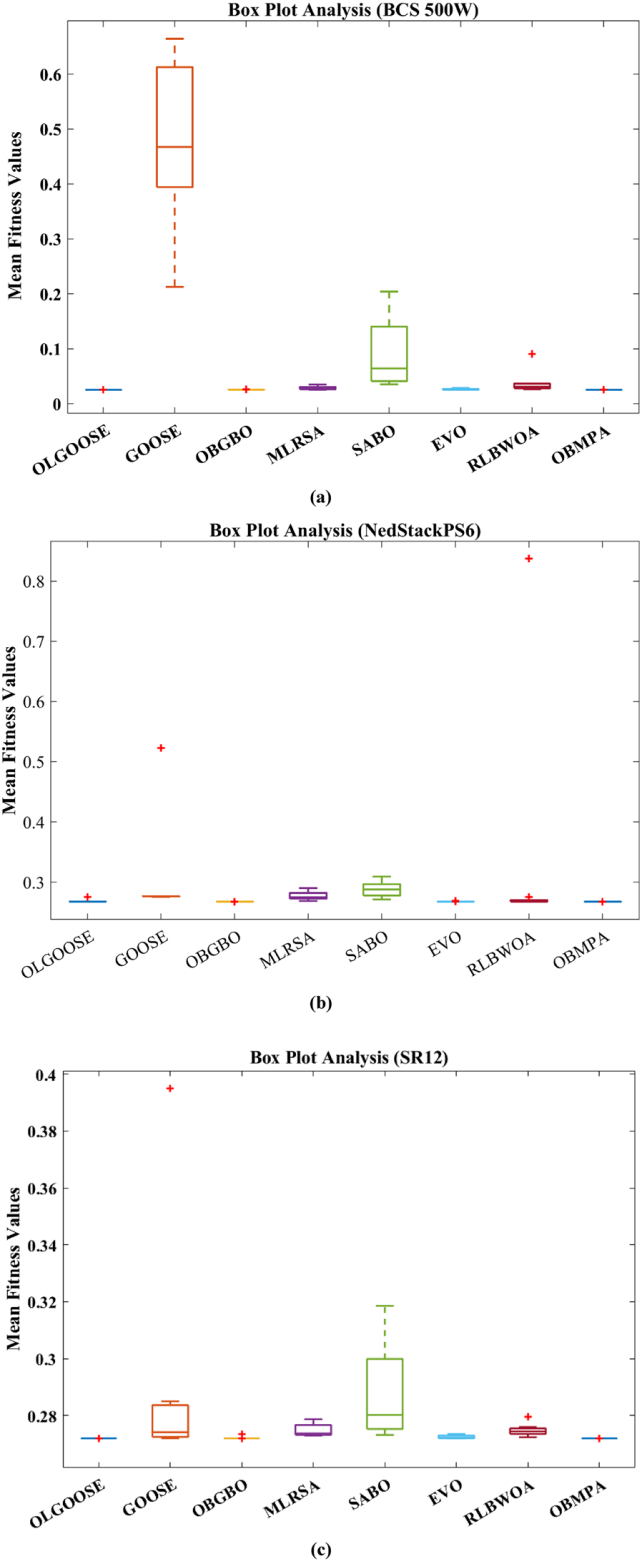
Box plot analysis; (**a**) BCS 500W, (**b**) NedStackPS6, (**c**) SR-12.

In terms of statistical distribution parameters, including median, range, and outliers, the boxplot analysis for the three FC models provides an understandable visual depiction of how the OLGOOSE method stacks up against alternative algorithms. As can be seen from the boxplot for BCS 500W in Fig. 10a, OLGOOSE performs more consistently and with less variability in voltage estimate due to its tightly packed quartile distribution. Additionally, the centre location of the box’s median line indicates that the data are symmetrically distributed around the median, which improves reliability. The fact that OLGOOSE’s performance shows no outliers suggests that it has remarkable control over extreme values, which reinforces its accuracy. The boxplot in Fig. 10b illustrates how OLGOOSE, in the NedStackPS6 model, maintains a small interquartile range akin to BCS 500W, indicating reliable and consistent voltage estimates over several runs. The fact that the median is comparatively lower than that of several algorithms and closely matches the experimental data suggests that the modelling was accurate. In addition to being near the box, which indicates reduced data dispersion, the minimum and maximum values also don’t have any notable outliers, suggesting consistent performance. The boxplot analysis displayed in Fig. 10c for the SR-12 model demonstrates the consistent estimation benefit provided by OLGOOSE and it becomes evident from its narrow box and shorter whiskers. The results demonstrate a highly symmetrical median, which does not deviate towards the quartiles, and it indicates that the estimates are not biased and are consistently centred. In summary, Fig. 10 provides strong evidence that OLGOOSE surpasses other methods in terms of parameter estimation in fuel cell models, delivering a higher level of reliability, consistency, and accuracy.

To thoroughly evaluate the performance of the various algorithms, we have tabulated the SSE and MAE for all case studies in Tables 5, 6, 7, 8, 9 and 10. These tables provide a comprehensive comparison of the results achieved by each algorithm. Upon examining Tables 5, 6, 7, 8, 9 and 10, it becomes evident that the proposed OLGOOSE algorithm and the OBMPA yield comparable results in terms of SSE and MAE. However, Table 4 highlights a significant difference in computational efficiency between the two. Specifically, the OBMPA requires four times more computational time than the OLGOOSE algorithm, making the latter more efficient. Furthermore, the reliability of the OLGOOSE algorithm surpasses that of the OBMPA. This is supported by the boxplot analysis, which shows that the OLGOOSE algorithm consistently produces more stable and reliable results across different datasets and case studies. When compared to the original GOOSE algorithm, the proposed OLGOOSE algorithm demonstrates superior performance metrics. Although the computational time for the OLGOOSE algorithm is slightly greater than that of the original GOOSE algorithm due to the incorporation of the orthogonal learning mechanism, this increase in computational time is justified by the significant improvements in accuracy and reliability. Moreover, when benchmarked against other algorithms, the OLGOOSE algorithm consistently outperforms them in terms of both performance metrics (SSE and MAE) and computational efficiency. The enhanced reliability of the OLGOOSE algorithm further solidifies its superiority. The orthogonal learning mechanism, despite adding some computational overhead, effectively enhances the algorithm’s capability to discover the solution space more carefully, leading to better overall performance. The proposed OLGOOSE algorithm offers a balanced approach, delivering high accuracy and reliability with reasonable computational efficiency. Its performance in terms of key metrics and reliability makes it a strong choice compared to other optimization algorithms evaluated in this study.

**Table 5:** SSE achieved by all algorithms for BCS 500W.

i_exp_ (A)	V_exp_ (V)	SSE							
		OLGOOSE	GOOSE	OBGBO	MLRSA	SABO	EVO	RLBWOA	OBMPA
0.6	29	7.71E−06	2.60E+00	5.54E−04	5.54E−04	2.08E−04	6.76E−03	4.33E−06	8.39E−06
2.1	26.31	1.65E−05	5.39E−01	7.00E−05	7.00E−05	6.97E−06	3.52E−04	5.11E−06	4.27E−05
3.58	25.09	1.26E−05	1.46E−01	1.96E−05	1.96E−05	2.20E−04	2.04E−04	3.30E−05	2.43E−04
5.08	24.25	2.13E−05	3.07E−02	6.17E−05	6.17E−05	2.84E−04	4.43E−04	4.98E−05	2.48E−04
7.17	23.37	2.93E−05	1.54E−05	9.81E−05	9.81E−05	2.65E−04	7.33E−04	6.60E−05	1.84E−04
9.55	22.57	2.21E−04	1.78E−02	3.69E−04	3.69E−04	4.84E−04	1.30E−03	3.12E−04	3.37E−04
11.35	22.06	2.28E−04	3.43E−02	2.42E−04	2.42E−04	2.35E−04	9.26E−04	2.15E−04	1.32E−04
12.54	21.75	7.42E−05	4.25E−02	1.53E−04	1.53E−04	1.01E−04	6.44E−04	1.44E−04	3.91E−05
13.73	21.45	2.27E−04	5.05E−02	2.18E−04	2.18E−04	1.07E−04	6.58E−04	2.25E−04	4.54E−05
15.73	21.09	1.05E−02	1.47E−02	9.89E−03	9.89E−03	1.15E−02	8.56E−03	9.63E−03	1.22E−02
17.02	20.68	2.22E−04	5.53E−02	2.84E−04	2.84E−04	4.53E−05	4.47E−04	3.58E−04	1.92E−05
19.11	20.22	1.21E−04	4.50E−02	1.59E−04	1.59E−04	4.40E−07	1.65E−04	2.50E−04	2.66E−06
21.2	19.76	2.20E−04	3.02E−02	1.42E−04	1.42E−04	1.32E−05	7.80E−05	2.60E−04	7.15E−06
23	19.36	3.63E−05	1.42E−02	4.10E−05	4.10E−05	9.34E−05	3.38E−06	1.28E−04	4.26E−05
25.08	18.86	4.18E−05	1.35E−03	3.61E−05	3.61E−05	5.55E−05	8.10E−06	1.31E−04	8.50E−07
27.17	18.27	2.23E−05	9.08E−03	7.40E−06	7.40E−06	2.71E−09	7.63E−05	6.59E−05	1.36E−04
28.06	17.95	1.10E−05	3.33E−02	1.84E−10	1.84E−10	6.58E−05	2.47E−04	2.56E−05	5.27E−04
29.26	17.3	5.07E−05	1.60E−01	2.09E−04	2.09E−04	8.00E−04	1.09E−03	1.18E−04	2.58E−03

**Table 6:** MAE achieved by all algorithms for BCS 500W.

i_exp_ (A)	V_exp_ (V)	MAE							
		OLGOOSE	GOOSE	OBGBO	MLRSA	SABO	EVO	RLBWOA	OBMPA
0.6	29	7.71E−06	1.54E−04	8.96E−02	1.31E−03	8.01E−04	4.57E−03	1.16E−04	1.61E−04
2.1	26.31	1.65E−05	2.26E−04	4.08E−02	4.65E−04	1.47E−04	1.02E−03	1.26E−04	3.63E−04
3.58	25.09	1.26E−05	1.98E−04	2.12E−02	2.46E−04	8.24E−04	5.68E−04	3.19E−04	8.67E−04
5.08	24.25	2.13E−05	2.57E−04	9.73E−03	4.36E−04	9.37E−04	1.71E−02	3.92E−04	8.74E−04
7.17	23.37	2.93E−05	3.01E−04	2.18E−04	5.50E−04	9.05E−04	1.50E−03	4.51E−04	7.54E−04
9.55	22.57	2.22E−04	8.12E−04	6.65E−03	1.07E−03	1.22E−03	2.00E−04	9.81E−04	1.02E−03
11.35	22.06	1.28E−04	6.29E−04	2.25E−02	8.65E−04	8.52E−04	2.69E−03	8.14E−04	6.38E−04
12.54	21.75	7.21E−05	4.70E−04	2.15E−03	6.86E−04	5.58E−04	2.41E−02	6.66E−04	3.47E−04
13.73	21.45	2.27E−04	6.26E−04	2.31E−02	8.20E−04	5.75E−04	2.43E−03	8.34E−04	3.74E−04
15.73	21.09	1.05E−02	5.68E−03	6.75E−03	5.53E−03	5.97E−03	5.14E−04	5.45E−03	6.13E−03
17.02	20.68	2.24E−04	8.06E−04	1.31E−02	9.36E−04	3.74E−04	1.17E−03	1.05E−03	2.43E−04
19.11	20.22	2.21E−04	6.10E−04	1.18E−02	7.01E−04	3.68E−05	7.13E−03	8.79E−04	9.05E−05
21.2	19.76	2.20E−04	6.08E−04	9.65E−03	6.62E−04	2.02E−04	4.91E−03	8.95E−04	1.49E−04
23	19.36	3.63E−05	2.35E−03	6.62E−03	3.56E−04	5.37E−04	1.02E−03	6.28E−04	3.63E−04
25.08	18.86	4.18E−05	2.59E−03	2.04E−03	3.34E−04	4.14E−04	1.58E−03	6.35E−04	5.12E−05
27.17	18.27	2.23E−05	2.62E−04	5.30E−03	1.51E−04	2.89E−06	4.85E−03	4.51E−04	6.48E−04
28.06	17.95	1.10E−05	1.84E−03	1.01E−02	7.53E−07	4.51E−04	8.73E−04	2.81E−04	1.28E−03
29.26	17.3	5.07E−05	3.96E−03	2.22E−02	8.04E−04	1.57E−03	1.83E−03	6.04E−04	2.82E−03

**Table 7:** SSE achieved by all algorithms for NedStackPS6.

i_exp_ (A)	V_exp_ (V)	SSE							
		OLGOOSE	GOOSE	OBGBO	MLRSA	SABO	EVO	RLBWOA	OBMPA
2.25	61.64	5.15E−01	5.33E−01	5.15E−01	5.20E−01	8.41E−01	5.15E−01	5.15E−01	5.15E−01
6.75	59.57	4.56E−02	4.94E−02	4.56E−02	4.68E−02	1.57E−01	4.56E−02	4.56E−02	4.56E−02
9.00	58.94	1.55E−03	1.41E−02	1.25E−02	1.31E−02	8.26E−02	1.25E−02	1.25E−02	1.25E−02
15.75	57.54	1.66E−03	1.52E−03	1.66E−03	1.55E−03	1.26E−02	1.66E−03	1.66E−03	1.66E−03
20.25	56.80	6.37E−03	6.59E−03	6.37E−03	6.23E−03	3.45E−03	6.37E−03	6.37E−03	6.37E−03
24.75	56.13	6.96E−03	7.70E−03	6.96E−03	6.92E−03	1.69E−03	6.96E−03	6.96E−03	6.96E−03
31.50	55.23	5.06E−03	6.32E−03	5.06E−03	5.14E−03	1.10E−03	5.06E−03	5.06E−03	5.06E−03
36.00	54.66	1.32E−03	4.39E−03	3.76E−02	1.53E−03	2.84E−03	1.45E−03	1.45E−03	1.45E−03
45.00	53.61	5.62E−04	7.36E−05	5.93E−02	4.70E−04	8.20E−03	5.62E−04	5.62E−04	5.62E−04
51.75	52.86	7.09E−03	4.41E−03	5.62E−04	6.64E−03	1.79E−02	7.09E−03	7.09E−03	7.09E−03
67.50	51.91	2.56E−01	3.44E−01	7.09E−03	2.26E−01	2.09E−01	6.57E−02	5.07E−02	1.91E−04
72.00	51.22	3.76E−02	4.71E−01	1.45E−03	3.93E−02	3.57E−02	2.08E−02	2.73E−01	2.24E−02
90.00	49.66	5.93E−02	7.15E−01	2.22E−01	6.18E−02	7.24E−02	1.91E−04	2.09E−01	1.35E−01
99.00	49.00	1.22E−02	1.58E−01	2.73E−01	1.44E−01	1.70E−01	1.35E−01	1.31E−02	1.93E−04
105.80	48.15	1.46E−02	2.04E−02	2.09E−01	1.59E−02	2.74E−02	2.22E−01	1.12E−01	5.07E−02
110.30	47.52	1.31E−02	8.81E−03	2.24E−02	1.19E−02	4.33E−03	3.76E−02	6.57E−02	1.12E−01
117.00	47.10	2.92E−03	5.29E−03	1.35E−01	3.51E−03	1.16E−02	1.31E−02	2.08E−02	2.09E−01
126.00	46.48	5.32E−03	5.97E−02	6.57E−02	5.47E−02	8.23E−02	2.92E−03	1.91E−04	2.08E−02
135.00	45.66	4.46E−02	4.96E−02	2.08E−02	4.65E−02	7.33E−02	2.09E−01	1.93E−04	6.57E−02
141.80	44.85	1.93E−04	4.89E−04	1.41E−01	3.21E−04	5.31E−03	1.12E−01	1.35E−01	1.31E−02
150.80	44.24	5.21E−02	4.24E−02	1.46E−02	5.22E−02	7.91E−02	5.24E−02	2.92E−03	2.73E−01
162.00	42.45	2.73E−02	3.75E−01	5.24E−02	2.71E−01	2.25E−01	4.46E−02	5.24E−02	4.46E−02
171.00	41.66	2.14E−01	3.14E−01	4.46E−02	2.08E−01	1.74E−01	1.93E−04	4.46E−02	2.22E−01
182.30	40.68	1.12E−01	1.19E−01	1.91E−04	1.13E−01	9.58E−02	5.93E−02	2.24E−02	3.76E−02
189.00	40.09	6.57E−02	7.13E−02	1.93E−04	6.67E−02	5.77E−02	1.41E−01	1.41E−01	5.93E−02
195.80	39.51	2.26E−02	2.39E−02	1.31E−02	2.17E−02	1.93E−02	1.46E−02	1.46E−02	2.92E−03
204.80	38.73	1.91E−04	5.25E−05	2.92E−03	8.47E−05	1.61E−05	2.24E−02	2.22E−01	5.24E−02
211.50	38.15	2.35E−02	2.21E−02	5.07E−02	2.07E−02	1.65E−02	5.07E−02	3.76E−02	1.41E−01
220.50	37.38	1.42E−01	1.43E−01	1.12E−01	1.29E−01	1.09E−01	6.57E−02	5.07E−02	1.91E−04

**Table 8:** MAE achieved by all algorithms for NedStackPS6.

i_exp_ (A)	V_exp_ (V)	MAE							
		OLGOOSE	GOOSE	OBGBO	MLRSA	SABO	EVO	RLBWOA	OBMPA
2.25	61.64	2.48E−02	2.52E−02	2.48E−02	2.49E−02	3.16E−02	2.48E−02	2.48E−02	2.48E−02
6.75	59.57	7.25E−03	7.41E−03	7.36E−03	7.46E−03	1.37E−02	7.36E−03	7.78E−02	7.36E−03
9.00	58.94	3.86E−02	5.21E−02	3.86E−02	3.94E−03	9.91E−03	3.86E−03	3.86E−03	3.86E−03
15.75	57.54	1.41E−03	1.34E−23	1.41E−03	1.36E−03	3.87E−03	1.41E−03	1.41E−02	1.41E−02
20.25	56.80	2.75E−03	2.80E−03	2.75E−02	2.72E−03	3.12E−02	2.75E−03	2.75E−03	2.75E−03
24.75	56.13	2.88E−03	3.03E−03	2.88E−03	2.87E−03	1.42E−03	2.88E−03	3.88E−02	2.88E−02
31.50	55.23	3.78E−03	2.74E−03	2.45E−02	2.47E−03	1.14E−02	2.45E−03	3.24E−03	3.78E−03
36.00	54.66	1.31E−04	1.69E−03	1.31E−03	1.35E−03	1.84E−03	1.31E−03	1.78E−02	1.31E−04
45.00	53.61	8.17E−04	2.96E−04	8.17E−04	7.47E−04	3.12E−03	8.17E−04	8.17E−04	8.17E−04
51.75	52.86	2.90E−03	2.29E−03	2.90E−03	2.81E−03	4.62E−02	2.90E−03	2.90E−03	3.01E−03
67.50	51.91	1.63E−02	1.70E−02	1.63E−02	1.64E−02	1.62E−02	1.63E−02	1.63E−02	1.63E−02
72.00	51.22	6.69E−03	7.48E−03	6.69E−03	6.84E−03	6.52E−03	6.69E−03	6.69E−03	6.69E−03
90.00	49.66	8.40E−03	9.22E−03	8.40E−03	8.57E−03	9.28E−03	8.40E−03	8.40E−03	8.40E−03
99.00	49.00	1.29E−02	1.37E−02	1.29E−02	1.31E−02	1.78E−02	1.29E−02	1.29E−02	1.29E−02
105.80	48.15	4.17E−03	4.92E−03	4.17E−03	4.35E−03	5.71E−03	4.17E−03	4.17E−03	4.17E−03
110.30	47.52	3.95E−03	3.24E−03	3.95E−03	3.77E−03	2.27E−02	3.95E−03	3.95E−03	3.95E−03
117.00	47.10	1.86E−03	2.51E−02	1.86E−03	2.04E−03	3.71E−03	1.86E−03	1.86E−03	1.86E−03
126.00	46.48	7.90E−03	8.43E−03	7.90E−03	8.06E−03	9.89E−03	7.90E−03	7.90E−03	7.90E−03
135.00	45.66	7.28E−03	7.68E−02	7.28E−03	7.44E−03	9.34E−03	7.28E−03	7.28E−03	7.28E−03
141.80	44.85	4.79E−04	7.63E−04	4.79E−04	6.18E−04	2.51E−03	4.79E−04	4.79E−04	4.79E−04
150.80	44.24	7.77E−03	7.89E−02	7.77E−03	7.88E−03	9.70E−03	7.77E−03	7.77E−03	7.77E−03
162.00	42.45	1.80E−02	1.81E−02	1.80E−02	1.80E−02	1.64E−02	1.80E−02	1.80E−02	1.80E−02
171.00	41.66	1.58E−02	1.60E−02	1.58E−02	1.57E−02	1.44E−02	1.58E−02	1.58E−02	1.58E−02
182.30	40.68	1.21E−02	1.19E−02	1.78E−02	1.16E−02	1.07E−02	1.21E−02	1.21E−02	1.216E−02
189.00	40.09	7.91E−03	9.21E−03	7.91E−03	8.90E−03	8.28E−03	7.91E−03	7.91E−03	7.91E−03
195.80	39.51	4.97E−03	5.33E−03	4.97E−03	5.08E−03	4.79E−03	4.97E−03	4.97E−03	4.97E−03
204.80	38.73	5.92E−04	2.50E−04	4.88E−04	3.17E−04	1.38E−04	5.92E−04	5.92E−04	5.92E−04
211.50	38.15	6.01E−03	5.13E−04	5.47E−03	4.96E−03	4.43E−03	6.01E−03	6.01E−03	6.01E−03
220.50	37.38	2.14E−02	1.31E−02	2.89E−02	1.24E−02	1.14E−02	2.14E−02	2.14E−02	2.14E−02

**Table 9:** SSE achieved by all algorithms for SR12.

i_exp_ (A)	V_exp_ (V)	SSE							
		OLGOOSE	GOOSE	OBGBO	MLRSA	SABO	EVO	RLBWOA	OBMPA
1.004	43.17	6.15E−02	5.87E−02	6.32E−02	5.61E−02	2.06E−01	8.15E−02	7.57E−02	6.15E−02
3.166	41.14	3.32E−04	1.45E−04	5.22E−04	1.41E−04	1.41E−02	2.51E−03	6.83E−04	3.32E−04
5.019	40.49	2.65E−01	2.72E−01	2.60E−01	2.68E−01	2.04E−01	2.38E−01	3.24E−01	2.65E−01
7.027	39.04	1.69E−02	1.89E−02	1.56E−02	1.68E−02	8.39E−03	1.17E−02	3.23E−02	1.69E−02
8.958	37.99	1.27E−04	3.58E−04	4.50E−05	6.62E−05	1.41E−04	3.78E−05	2.50E−03	1.27E−04
10.97	37.08	7.65E−04	1.28E−03	5.81E−04	4.96E−04	2.42E−04	2.20E−04	2.69E−03	7.65E−04
13.05	36.03	6.45E−03	5.16E−03	6.78E−03	7.64E−03	7.17E−03	7.88E−03	5.26E−03	6.45E−03
15.06	35.19	2.45E−05	1.51E−05	2.64E−05	1.74E−04	2.02E−05	9.22E−05	1.68E−04	2.45E−05
17.07	34.07	3.58E−02	3.25E−02	3.51E−02	3.92E−02	3.46E−02	3.63E−02	4.50E−02	3.58E−02
19.07	33.02	7.53E−02	7.03E−02	7.30E−02	8.01E−02	7.32E−02	7.46E−02	9.61E−02	7.53E−02
21.08	32.04	5.59E−02	5.16E−02	5.29E−02	5.97E−02	5.47E−02	5.46E−02	7.96E−02	5.59E−02
23.01	31.2	1.51E−03	8.75E−04	9.23E−04	2.07E−03	1.56E−03	1.25E−03	8.21E−03	1.51E−03
24.94	29.8	1.05E−01	9.96E−02	9.91E−02	1.09E−01	1.09E−01	1.04E−01	1.43E−01	1.05E−01
26.87	28.96	2.36E−03	3.28E−03	3.47E−03	2.13E−03	1.17E−03	2.39E−03	2.00E−08	2.36E−03
28.96	28.12	4.49E−01	4.59E−01	4.61E−01	4.50E−01	4.13E−01	4.42E−01	4.03E−01	4.49E−01
30.81	26.3	1.00E−01	1.04E−01	1.03E−01	1.02E−01	7.39E−02	9.20E−02	9.24E−02	1.00E−01
32.97	24.06	6.57E−03	7.41E−03	5.81E−03	7.93E−03	7.11E−05	2.84E−03	1.28E−02	6.57E−03
34.9	21.4	1.48E−01	1.46E−01	1.65E−01	1.39E−01	2.45E−01	1.88E−01	8.31E−02	1.48E−01

**Table 10:** MAE achieved by all algorithms for SR12.

i_exp_ (A)	V_exp_ (V)	MAE							
		OLGOOSE	GOOSE	OBGBO	MLRSA	SABO	EVO	RLBWOA	OBMPA
1.004	43.17	1.38E−02	1.35E−02	1.40E−02	1.32E−02	2.52E−02	1.59E−02	1.53E−02	1.38E−02
3.166	41.14	1.01E−03	6.68E−04	1.27E−03	6.59E−04	6.60E−03	2.78E−03	1.45E−03	1.01E−03
5.019	40.49	2.86E−02	2.90E−02	2.83E−02	2.88E−02	2.51E−02	2.71E−02	3.16E−02	2.86E−02
7.027	39.04	7.23E−03	7.63E−03	6.95E−03	7.21E−03	5.09E−03	6.01E−03	9.99E−03	7.23E−03
8.958	37.99	6.26E−04	1.05E−03	3.73E−04	4.52E−04	6.59E−04	3.41E−04	2.78E−03	6.26E−04
10.97	37.08	1.54E−03	1.99E−03	1.34E−03	1.24E−03	8.65E−04	8.24E−04	2.88E−03	1.54E−03
13.05	36.03	4.46E−03	3.99E−03	4.57E−03	4.86E−03	4.70E−03	4.93E−03	4.03E−03	4.46E−03
15.06	35.19	2.75E−04	2.16E−04	2.86E−04	7.32E−04	2.49E−04	5.33E−04	7.20E−04	2.75E−04
17.07	34.07	1.05E−02	1.00E−02	1.04E−02	1.10E−02	1.03E−02	1.06E−02	1.18E−02	1.05E−02
19.07	33.02	1.52E−02	1.47E−02	1.50E−02	1.57E−02	1.50E−02	1.52E−02	1.72E−02	1.52E−02
21.08	32.04	1.42E−03	2.26E−02	2.28E−02	1.36E−02	1.24E−02	1.45E−02	1.57E−02	1.42E−02
23.01	31.2	2.16E−03	1.64E−03	1.69E−03	2.53E−03	2.19E−03	1.97E−03	5.03E−03	2.16E−03
24.94	29.8	1.80E−02	1.75E−02	1.75E−02	1.83E−02	1.84E−02	1.79E−02	2.10E−02	1.80E−02
26.87	28.96	2.68E−03	4.18E−03	4.33E−03	2.56E−03	1.90E−03	2.72E−03	7.86E−06	3.70E−03
28.96	28.12	3.72E−02	3.77E−02	3.77E−02	3.73E−02	3.57E−02	3.69E−02	3.53E−02	3.72E−02
30.81	26.3	1.76E−02	1.79E−02	1.78E−02	1.78E−02	1.51E−02	1.68E−02	1.69E−02	1.76E−02
32.97	24.06	4.71E−03	4.78E−03	5.34E−03	4.95E−03	5.52E−04	2.96E−03	6.29E−03	5.50E−03
34.9	21.4	2.14E−02	2.12E−02	2.26E−02	2.07E−02	2.75E−02	2.41E−02	1.60E−02	2.14E−02

**Fig. 11:**
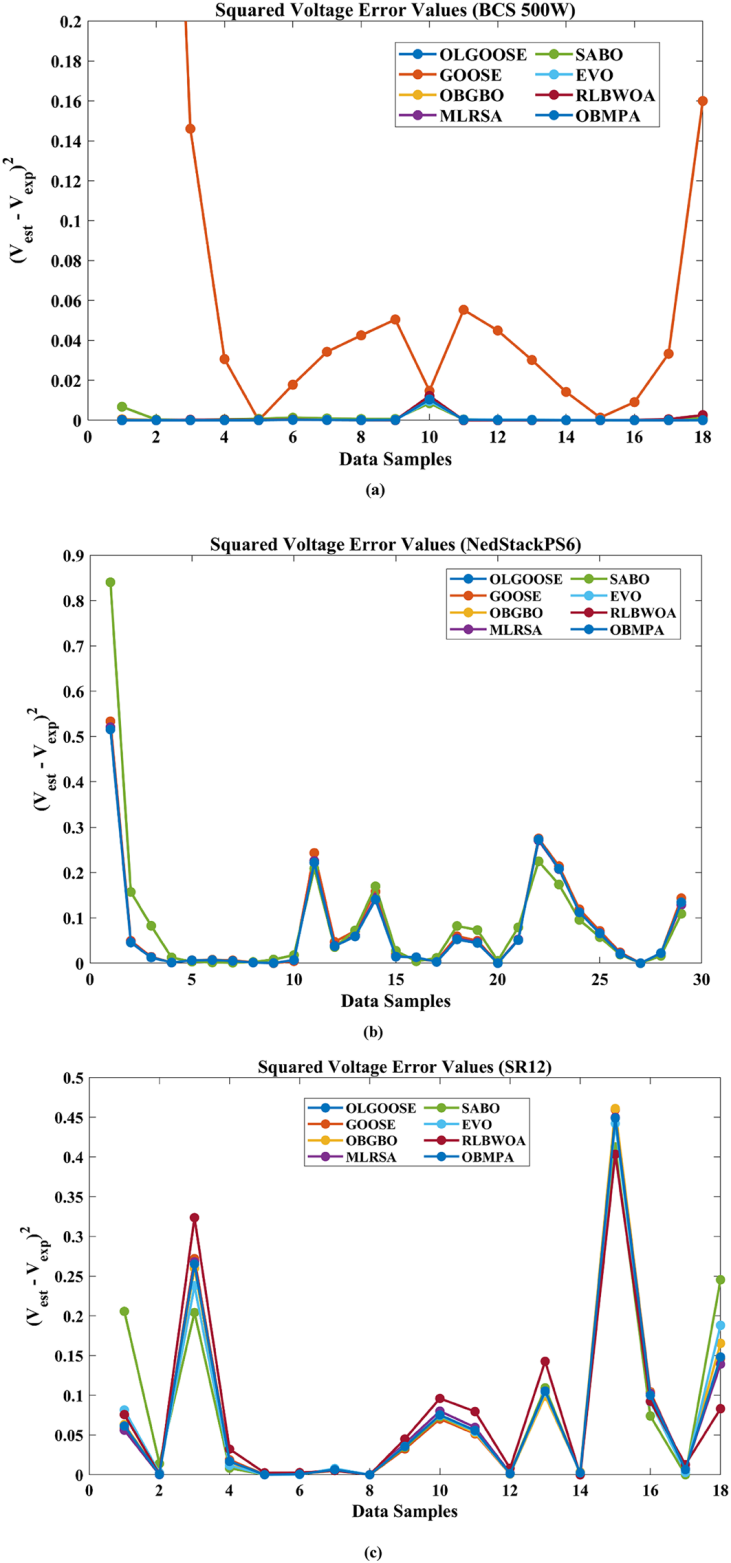
Error rate achieved by algorithms, (**a**) BCS 500W, (**b**) NedStackPS6, (**c**) SR-12.

In order to visualize the error metric, Fig. 11 presents error plots that comprehensively display the performance of various algorithms, including OLGOOSE, across three different FC models. The error plots in Fig. 11 serve as a visual tool to measure and compare the magnitude of errors generated by each algorithm under varying operational conditions. By examining Fig. 11, one can distinguish the consistency and accuracy of each algorithm. Specifically, Fig. 11 highlights that the proposed algorithm exhibits the smallest errors consistently across all tested conditions and models. Figure 11 clearly defines which algorithm, including OLGOOSE, manages to maintain precision across a diverse set of conditions and models, effectively highlighting its superiority in terms of both accuracy and robustness.

### Further discussions

OLGOOSE’s superior performance across all FC models can be attributed to several key features and enhancements integrated within the algorithm, which collectively improve its accuracy, robustness, and efficiency. The introduction of orthogonal learning in OLGOOSE is a fundamental enhancement that significantly contributes to its superior performance. Orthogonal learning helps in diversifying the search patterns of the algorithm, ensuring that the solution space is explored more comprehensively. By using orthogonal vectors, OLGOOSE can efficiently escape local optima and explore multiple dimensions of the problem space simultaneously, reducing the risk of convergence on sub-optimal solutions.

OLGOOSE strikes an optimal balance between global and local search strategies. The orthogonal vectors facilitate broad, global explorations initially, which is crucial for identifying promising regions within a vast search space. As the algorithm progresses, it gradually shifts towards more refined local searches, focusing in on the best solutions with high accuracy. This dynamic adjustment between exploration and exploitation phases allows OLGOOSE to maintain high accuracy and adaptability across different operational conditions and FC models. In the FC models, precise parameter estimation is critical for modelling the complex chemical and physical interactions accurately. OLGOOSE’s algorithmic structure is particularly adept at tuning these parameters to reflect the true behaviour of the system under study. By effectively minimizing the error metrics such as MAE, SSE, and RMSE, OLGOOSE demonstrates its capability to tune parameters that result in models which closely mimic real-world data. The stochastic nature of metaheuristic algorithms often leads to variability in performance. However, OLGOOSE is designed to offer more consistent results across multiple runs. This consistency is evidenced by the smaller standard deviations in error metrics, suggesting that OLGOOSE not only finds better solutions but does so reliably over successive iterations. Such reliability is particularly valuable in practical applications where repeatability of results is crucial. Despite its complex internal mechanisms, OLGOOSE is optimized for computational efficiency. OLGOOSE’s ability to quickly converge to optimal solutions without excessive computational overhead makes it suitable for real-time and scalable applications.

Fuel cells come in various types and configurations, each with unique characteristics and operational dynamics. OLGOOSE’s performance across multiple FC models suggests that it has inherent flexibility and adaptability, capable of adjusting its optimization strategy to suit different types and sizes of data sets and models. In summary, OLGOOSE’s integration of orthogonal learning, balanced search capabilities, robust parameter optimization, consistent and reliable performance, computational efficiency, and adaptability are key reasons behind its superior performance across different FC models. These features not only enhance the precision of the model but also ensure that it can be reliably used in diverse applications, reaffirming its status as a preferred algorithm in the field of optimization. The OLGOOSE algorithm’s flexibility allows for its application across different PEMFC models, requiring the estimation of various parameters unique to each model. This adaptability ensures that the approach remains robust and applicable to a wide range of modelling frameworks.

## Conclusions

The proposed OLGOOSE algorithm is combined with the orthogonal learning mechanism and it demonstrated superior performance in optimizing parameters for three different fuel cell models. The superior performance of the OLGOOSE algorithm is due to the orthogonal learning mechanism, which effectively balances the exploration and exploitation phases of the original GOOSE algorithm. The investigation in this study illustrates the effectiveness of the OLGOOSE in terms of accuracy and efficiency, outperforming conventional metaheuristic algorithms across various parameters such as RMSE, SSE, MAE, RT, and FRT. The strong mechanism of OLGOOSE is the foundation of its strength and allows for precise and reliable estimation of the fuel cell behaviour. The stability and consistency demonstrated in several experimentations, statistical parameters, and statistical tests make the proposed algorithm a reliable tool for fuel cell parameter estimation. OLGOOSE addresses the challenge of parameter optimization by enhancing the operational efficiency of fuel cell systems and achieves a prominent balance between comprehensive search capabilities and precise improvements. As a result, this is highly advantageous for academics and practitioners who aim to advance the boundaries of energy technology and optimization.

In the future, OLGOOSE may expand its application to include additional complex systems such as battery management and renewable energy sources. By integrating OLGOOSE with machine learning predictions and real-time data processing, it has the potential to become a valuable tool in the energy market. This future extension would enhance its practicality in everyday situations. In addition, advancements in computational methods could enhance its effectiveness by enabling faster and more adaptable procedures that are ideal for handling larger and more complicated datasets. These additional paths are expected to enhance the usefulness of OLGOOSE and yield discoveries in the disciplines of energy system optimization and other related areas.

## Data Availability

This study does not use any new data and existing data will be provided upon valid request.
